# Novel feature-based method for multi-modal biomedical image registration compared to intensity-based technique

**DOI:** 10.1038/s41598-025-12862-2

**Published:** 2025-08-01

**Authors:** Mohammad Javad Shojaei, Lichen Yang, Kazem Shojaei, Jeerapat Doungchawee, Richard W. Vachet

**Affiliations:** 1 Department of Materials, Imperial College London, London, UK; 2 School of Computing, Newcastle University, Newcastle upon Tyne, UK; 3 School of Medicine, Tehran University of Medical Sciences, Tehran, Iran; 4 Department of Chemistry, University of Massachusetts Amherst, Amherst, USA

**Keywords:** Computational biology and bioinformatics, Mathematics and computing

## Abstract

Multimodal image registration plays a crucial role in biomedical research, enabling the integration of complementary information from different imaging techniques. We present a novel feature-based approach for multimodal image registration, alongside traditional intensity-based methods. Our method, inspired by SPP-net architecture, employs multi-level feature extraction for robust image alignment. Additionally, we perform t-SNE dimensionality reduction on the MALDI-MSI dataset to enhance feature discrimination and visualization. We evaluated both approaches using datasets from the ANHIR Grand Challenge and mass spectrometry imaging modalities (LA-ICP-MS and MALDI-MSI). The proposed feature-based method achieved comparable accuracy to optimized intensity-based approaches, with Dice Coefficients of 0.95 for ANHIR samples (e.g., COAD_05) and 0.97 for mass spectrometry data, while requiring approximately 50% less computational time. Quantitative evaluation through Mutual Information metrics and Hausdorff Distance demonstrated high registration accuracy across different tissue types and imaging modalities. These results establish our feature-based approach as an efficient alternative to traditional intensity-based methods for multimodal image registration in biomedical applications.

**Supplementary Information:**

The online version contains supplementary material available at 10.1038/s41598-025-12862-2.

## Introduction

A profound understanding of diseases, such as cancer, demands a multifaceted and comprehensive analysis that leverages multiple biomedical imaging modalities to enable accurate diagnosis, effective treatment planning, and the discovery of novel therapeutic approaches^1,2^. Biomedical imaging plays a crucial role in biomedical research, aiding in diagnosing, treating, and monitoring various diseases, including cancer^3,4^. However, no single modality provides a complete picture; thus, integrating data from different sources has become increasingly critical in both clinical and research settings. This is where image registration comes in, which is the process of aligning two or more images of the same sample acquired from different modalities and machines or taken at different times or from adjacent serial Sects.^5–7^. Multimodal image registration allows researchers and clinicians to correlate structural and functional data, improving diagnostic accuracy and providing deeper insight into disease progression^8–10^.

Methods to visualize different morphological, cellular, biochemical, and elemental features in tissues are important for characterizing healthy and diseased regions. These imaging and analysis techniques play a critical role in understanding disease progression, identifying diagnostic markers, and developing targeted therapies. Among the most widely used approaches are Hematoxylin and Eosin (H&E) staining and mass spectrometry (MS) imaging, which offer complementary perspectives on tissue architecture and molecular composition. H&E staining is a widely used technique for identifying histological features of tissue samples, such as cell morphology and tissue architecture, which can identify regions of healthy and diseased tissue and providing insight into underlying disease mechanisms^11,12^. However, while it excels at visualizing structural aspects of tissues, it lacks the ability to detect or quantify specific molecular biomarkers. To complement this limitation, molecular imaging techniques like mass spectrometry (MS) imaging have emerged as powerful tools for exploring the biochemical and elemental landscape of tissues. MS imaging, on the other hand, provides valuable data on the distribution of biomolecules and elements in tissue sections, which can be correlated to specific molecular markers or used to understand the impact of endogenous dyshomeostasis or exogenous exposure within biological systems^13–15^. This modality is particularly valuable for studying metabolic alterations, identifying disease-specific biomarkers, and mapping drug distributions within tissues. For example, in oncology research, MS imaging can reveal metabolic signatures of tumor regions that may not be distinguishable by morphology alone. Additionally, it allows the investigation of dysregulated elemental distributions, such as altered iron or calcium levels, which are associated with a range of diseases including neurodegeneration and cancer.

By integrating different imaging datasets through image registration, researchers can co-localize and combine the data from each modality, gaining a more thorough understanding of the molecular and cellular characteristics of the tissue^16–18^. This process allows complementary information—such as cellular morphology from histological staining and molecular or elemental distribution from mass spectrometry or other imaging techniques to be examined within the same spatial context. As a result, the combined dataset offers a more holistic view of the molecular and cellular landscape of tissues than would be possible with a single modality alone^19,20^. For instance, image registration can help link morphological abnormalities to specific biochemical or molecular changes, aiding in the identification of novel diagnostic biomarkers or therapeutic targets. Such detailed spatial correlation is particularly valuable in diseases like cancer, where tumor microenvironments often exhibit complex interactions between different cell types and signaling molecules. By precisely overlaying structural and functional data, researchers can explore these interactions with greater accuracy and uncover spatial patterns that may be critical for disease characterization.

However, it is essential to consider that each imaging modality may have inherent limitations and potential biases that must be carefully considered in interpreting the results^21–23^. These differences can introduce alignment errors, registration artifacts, or interpretation biases if not carefully accounted for. Furthermore, the sample preparation protocols may differ between modalities, potentially affecting the biological integrity or compatibility of the datasets. Nevertheless, the integration of these diverse datasets significantly advances our understanding of disease biology and paves the way for potential breakthroughs in diagnosis and treatment of diseases like cancer^24^.

In this study, we explored two main approaches for image registration: Feature-based and Intensity-based. More detailed descriptions of intensity-based approaches are provided in the supplementary information file. The Feature-based approach transforms features extracted from the images into the same coordinate system^25,26^. On the other hand, Intensity-based methods, also known as direct methods, consider the intensity values of pixels to find the optimal transformation that makes the images as similar as possible^27,28^. Features could be lines, corners, or other distinct structures within the image^29,30^. The main advantage of Feature-based methods is their efficiency, as they only need to process the features rather than the entire image. However, these methods rely heavily on the quality of feature detection and matching^31^. Traditional feature extraction methods such as FAST^32^ ORB^33^ and SIFT^34^ are well known for their computational efficiency and are widely used in robotic mapping and visual 3D reconstruction^35–38^. However, these applications typically involve image data acquired from the same imaging device, either continuously or within a short time span, or from similar types of devices. This limitation reduces the effectiveness of such methods when applied to multi-modal images, particularly in the context of medical imaging. To address feature mismatches in image registration tasks, some techniques combine multiple feature detection methods to enhance feature matching precision. Engin el al^37^ had demonstrated the combination of SIFT and SURF achieved high accuracy on chest X-ray images. Yanhai et al.^39^ combined abilities of robust feature detection from SURF and region representation from FREAK descriptor. Goncalves et al.^38^ improved accuracy of point pairs by combining image segmentation with SIFT. Additionally, some researchers employ preprocessing or post-processing techniques to remove outliers and improve the quality of feature matching. Li, D et al.^40^ implemented an angle constraint to feature points to remove false matching points. A teaching learning-based optimization is proposed by Arora^41^ to optimal rigid transformation on Whole Brain ATLAS and KAGGLE datasets. Samantaray et al.^42^ validated the combination of SURF and BRISK algorithm for feature detection and description, then further improved matching accuracy by GMS algorithm and RANSAC. To enhance the representational ability of image features and reduce the computational cost of image registration algorithm in multi-modal medical images, we propose a novel feature extraction method tailored for multi-modal medical image registration. Our method enables robust feature detection and matching across different imaging modalities by identifying features with high local intensity variation and producing consistent feature descriptions across modalities.

After exploring both feature-based and intensity-based image registration methods, we conducted a series of carefully designed experiments to determine the optimal conditions for each approach. These experiments aimed to assess the robustness, efficiency, and accuracy of the methods across a range of scenarios. Once optimal parameters were established, we employed quantitative evaluation metrics, including the Dice coefficient and Hausdorff distance, to systematically compare their performance. This comparative analysis allowed us to identify the methodology that delivered higher registration accuracy. The rigorous and methodical nature of this evaluation reinforces the reliability and validity of our proposed feature-based pipeline, underscoring its contribution to the overall robustness of the study. This our method is highly relevant to cancer research, where 2D imaging modalities such as H&E, mass spectrometry (MS), laser ablation-coupled plasma-ion mobility spectrometry (LA-ICP-IMS), and imaging mass cytometry (IMC) are widely used.

The key contributions of this work for the registration of multimodal medical images are outlined as follows:

- A novel, unsupervised feature extraction method for multi-modal medical image registration, which leverages hierarchical average pooling operations to identify features with high local intensity variation.
- Extensive experiments validating that the proposed feature extraction method outperforms ORB and SIFT in the context of multi-modal medical image registration.
- Comprehensive quantitative analysis comparing the performance of the feature extraction-based image registration method with a traditional intensity-based registration method.

## Materials

In this study, we utilized publicly available multimodal datasets from the ANHIR Grand Challenge platform to evaluate the performance of our proposed Feature-based image registration method, as well as an additional dataset from matrix-assisted laser desorption/ionization MS imaging (MALDI-MSI) and laser ablation inductively coupled plasma MS imaging (LA-ICP-MSI) measurements. The ANHIR platform provides a diverse collection of medical imaging datasets specifically designed for benchmarking image registration algorithms. We selected six datasets from the platform, each representing distinct tissue types and imaging characteristics, to ensure comprehensive testing and robustness of our approach. More details are provided in Table 1.

**Table 1:** Summary of the datasets used in this study, including their descriptions and unique characteristics, highlighting the diversity in tissue types and imaging features. These datasets were obtained from the ANHIR grand challenge platform and were utilized to evaluate the proposed image registration method.

Datasets	Description
COAD_05	Images of colon adenocarcinoma tissue, characterized by complex cellular patterns and challenging morphological features.
Breast_4	Histological images of breast tissue, which present significant structural and intensity variations.
Kidney_4	Histological images of kidney tissue, providing a mixture of regular and irregular structural features.
Lung-lesion_1	Images of lung lesion samples, with high variability in intensity and texture across different regions.
Mammary-gland_1	Tissue sections from mammary glands, presenting subtle and intricate morphological details.
Mice-kidney_1	Images of mouse kidney tissue, offering a smaller scale and finer anatomical structures for analysis.

Each dataset consists of fixed and moving image pairs representing corresponding sections of tissue, often captured under different staining protocols or imaging modalities. These variations make the datasets suitable for evaluating both the accuracy and robustness of registration methods, particularly in challenging cross-modal scenarios.

### Materials and tissue preparation for MALDI-MSI and LA-ICP-MS experiments

Liver tissues were collected from Balb/c mice that were administered tail-vein injections of gold nanoparticles (AuNPs) and sacrificed six days after injection. These samples are part of a dataset previously published^43^. Following the collection, the liver tissues were flash-frozen and kept at − 80 °C until sectioned for imaging. Flash-frozen liver tissues were cryo-sectioned to 12 μm using a LEICA CM1850 cryostat. Adjacent tissue sections were thaw-mounted on indium tin oxide (ITO)-coated glass for MALDI-MSI and glass slides for LA-ICP-MSI experiments.

### LA-ICP-MSI experiment

LA-ICP-MS images of ^197^Au, ^66^Zn, and ^57^Fe were acquired on a CETAC LSX-213 G2 laser ablation system coupled with a Perkin Elmer NexION 300X ICP-MS instrument. Tissues were ablated via line scanning as described in previous work^44^. Data acquisition was performed at 50 μm resolution images with the following laser parameters: 50 μm spot size, 20 μm/s scan rate, 3.65 J laser energy, 10 Hz laser frequency, and a 10 s shutter delay. The helium carrier gas from the laser ablation system was set to 0.6 L/min.

### MALDI-MSI experiment

MALDI-MSI experiments were performed using 2,5-dihydroxybenzoic acid (2,5-DHB) as a matrix. Matrix deposition was performed by sublimation using a home-built sublimation apparatus. Briefly, 200 mg of the matrix was spread over the bottom chamber, then the temperature was set at 140 °C and the vacuum pressure was 7 mTorr for 9 min to allow matrix deposition. Data acquisition was performed on a Bruker UltrafleXtreme MALDI TOF/TOF at 50 μm resolution over a m/z range of 200 to 2000.

## **Methods**

### Image pre-processing

**Fig. 1:**
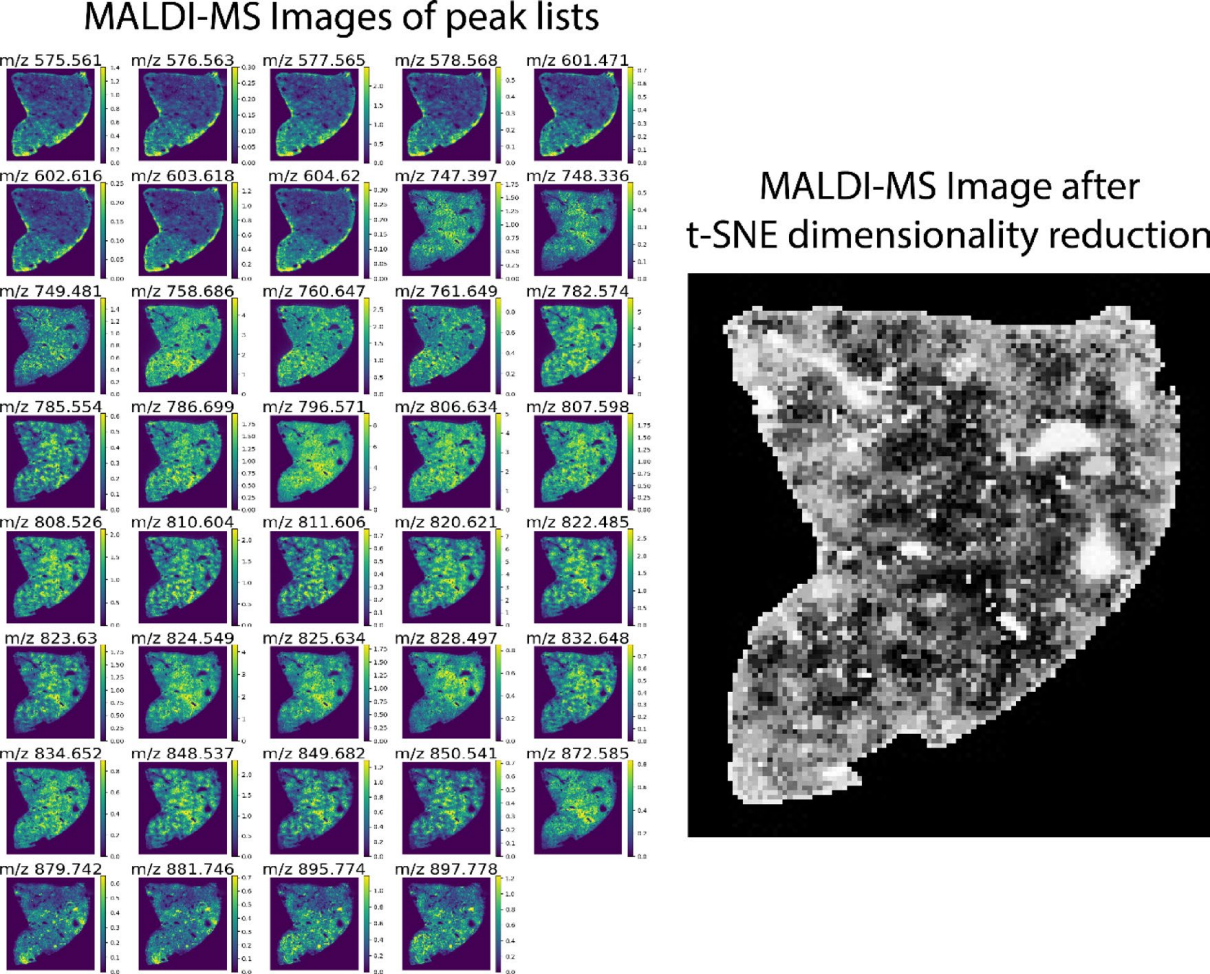
A collection of MALDI-MS images depicting the spatial distribution of various ions (m/z values) within a tissue sample. The rightmost image illustrates the result of t-SNE dimensionality reduction, which clusters similar ion distributions together.

We utilized the h5py Python package to read LA-ICP-MSI datasets saved in h5 format. For H&E, we employed Qupath software to read, extract, and export the dataset^45^. LA-ICP-MS images were reconstructed, analyzed, and segmented using RecSegImage-LA^46^. MALDI-MS images were normalized and saved as imzML files using SCiLS Lab software (Bruker, Daltonics). Peak lists and mass tolerance were selected in SCiLS Lab and imported into Python via a text file. Images for these selected ions were then produced using the pyimzML parser. For MALDI-MSI data, dimensionality reduction was achieved by applying the t-stochastic non-linear embedding (t-SNE) method from the sci-kit-learn Python library^47^resulting in an image representation of the dataset as shown in Fig. 1. Hotspot removal was implemented in MALDI-MS and LA-ICP-MS images by adjusting intensities above the 0.99 quantile to match the 0.99 quantile value^48^. Some pair modalities have a 90◦ difference in direction. We applied an initial 90◦ rotation before registration.

### Image registration

Image registration is a process that involves aligning two or more images to a common coordinate system. In mathematical terms, image registration seeks to find a transformation function that, when applied to the moving image, aligns it with the fixed image. The two primary approaches for image registration are Feature-based and Intensity-based methods, and we will discuss them in more detail below. A detailed description of Intensity-Based Method is presented is Supplementary Information.

### Feature-based method

**Fig. 2:**
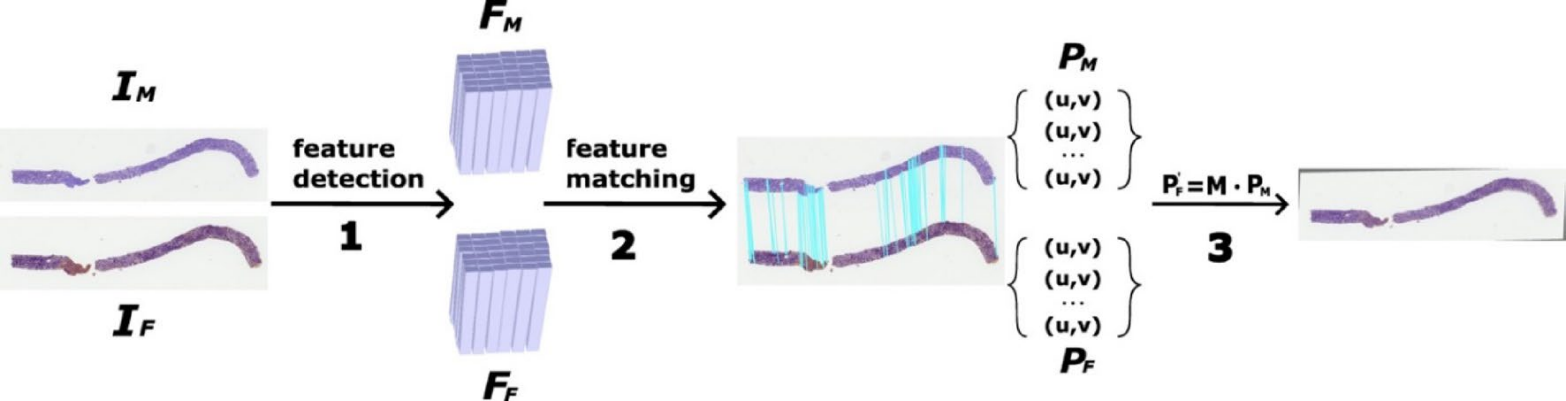
Workflow of the feature-based registration process. (1) Features are detected based on point-wise intensity calculations. (2) The most similar features from the two modalities are paired, with blue lines linking the feature centers in the modality images. (3) The transformation matrix between the two images is estimated using the coordinates of the paired points.

As shown in Fig. 2, the Feature-based registration pipeline consists of three phases. Firstly, in the feature detection phase, a multi-scale pooling operation computes feature descriptors pixel-wise on the input images (${I}_{M}$ and ${I}_{F}$) to produce individual feature maps (${F}_{M}$ and ${F}_{F}$). Key features are then sorted and selected based on the top N magnitudes of their feature vectors. Secondly, in the feature matching phase, corresponding key features in the fixed image are matched to features in the moving image using the closest Euclidean distance in feature space. High-confidence feature pairs are filtered through a perspective transformation between the two image planes. Finally, the Affine transformation matrix $M$ is derived through a least-squares calculation based on the key point locations (${P}_{M}$ and ${P}_{F}$) in the moving image and the fixed image, using the matched feature pairs.

**Fig. 3:**
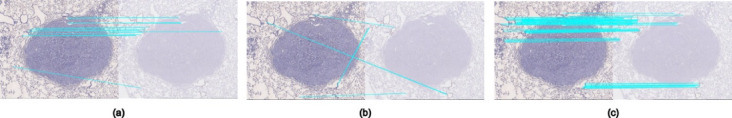
(**a**–**c**) show the results of feature matching with filtering using ORB, SIFT and our proposed feature-based method. (**a**) An incorrect match is shown at the bottom of the image. (**b**) Feature matching is sparse and inaccurate.

**Fig. 4:**
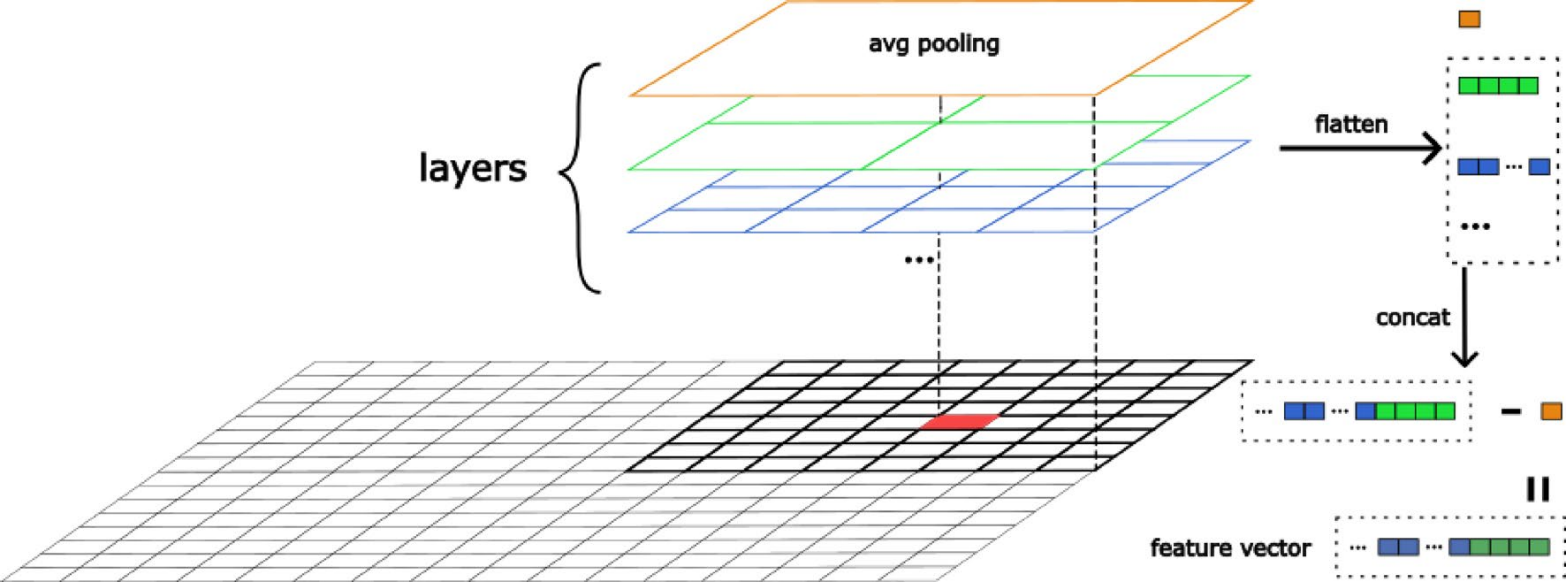
The diagram of the multi-layer feature extraction method. The fixed-length feature of the center pixel coordinate (red) is computed using an average pooling operation within each window block and then concatenated.

In this study, we tested two commonly used feature extraction methods in computer vision as control methods. They are ORB (Oriented FAST and Rotated BRIEF) and SIFT (Scale-Invariant Feature Transform). ORB is a method that combines FAST and BRIEF algorithms to detect and describe corners or interest points in images by comparing the brightness and intensity values of pixels within and around the points^49^. SIFT is a method that creates a scale space and computes the Difference of Gaussian (DoG) to locate and describe key points in images that are invariant to scale and rotation^50^. However, in our case, these two feature detection and matching methods are highly sensitive to modality changes, resulting in suboptimal matching outcomes for registration as shown in Fig. 3. Matched feature pairs from the two modalities should be visually similar. The two ends of each blue line in Fig. 4 exhibit a similar texture and a relatively consistent position within the tissue, indicating good feature matching results. A higher number of well-matched feature pairs leads to a more stable and reliable transformation calculation between the two images.

### Feature detection

The feature detection approach that we propose draws inspiration from the pyramid network structure and multi-level pooling operations of SPP-net (Spatial Pyramid Pooling in Deep Convolutional Networks)^51^. This method extracts features from image regions that have high intensity variations and uses average pooling to aggregate the relative intensity values at different scales to obtain a fixed-length feature vector. The use of average pooling operations in this method is employed to mitigate the impact of image noise on features. Because the feature extraction method is learnable parameter free and the input is a raw image, in which the max value is tent to be signal noise.

Figure 4 illustrates the structure of the feature computation model. The grey grid represents the input image, and the black-shaded area indicates the working window. In this diagram, each block, equally divided in a layer, represents an average pooling operation (referred to as “avg pooling”), which computes the mean intensity value of an image block as $\frac{1}{W\times\:W}{\sum\:}_{v}^{H}{\sum\:}_{u}^{W}{I}_{u,v}$. The model consists of multiple layers $L$, with each layer $l\:\in\:L$ containing ${2}^{l-1}$ pooling operations. The pooling values from all layers are flattened and concatenated into a single feature vector. To focus on capturing intensity variations within the working window and ignore exposure changes across the image, the average value of the top layer is removed and subtracted from the feature vector. Ultimately, the resulting feature vector serves as the representation for the center point (highlighted in red in the diagram).

**Fig. 5:**
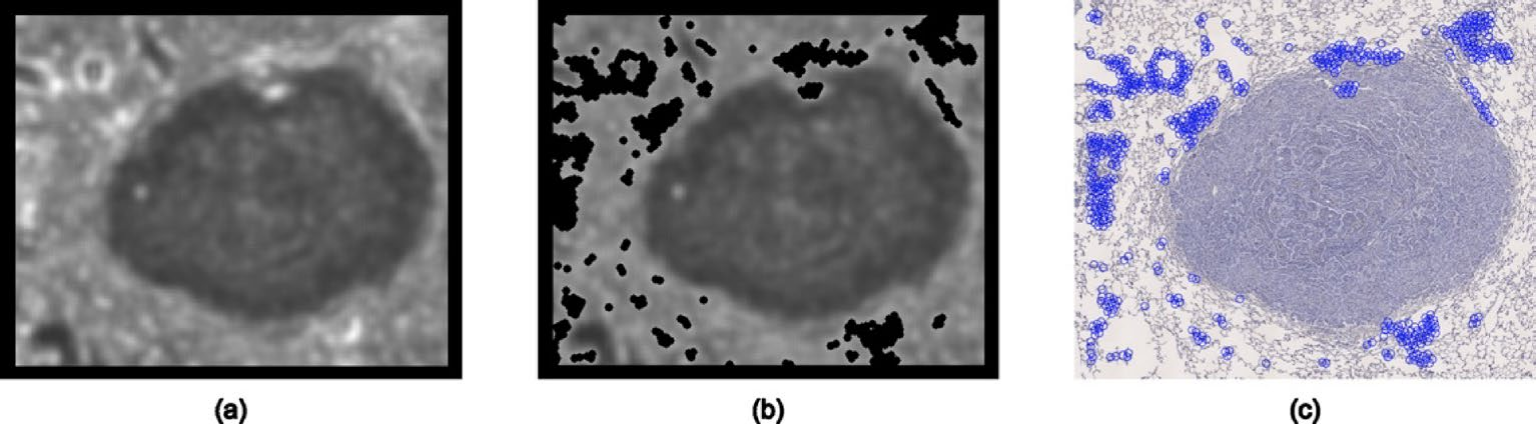
The process of feature selection. (**a**) Feature magnitude map. (**b**) After a feature is selected, a black circle is masked on the feature position. (**c**) Selected features (blue circles).

The process of feature selection is shown in Fig. 5. Brighter areas on the map (Fig. 5a) indicate regions with higher intensity variation in the image, corresponding to more confident features. The black border represents the out-of-range area for feature detection, with a width equal to half the working window size. Using this magnitude map, key features are selected in descending order based on the brightness of the points. To control the feature density, a distance regulation (${\mathcal{D}}ist\left( {F_{i} } \right) > D$) is applied to each feature position during the selection process as shown in Eq. 1.

1 $${\mathcal{D}}ist\left( {F_{i} } \right) = \mathop {\min }\limits_{{x \in \left\{ {0 \ldots i - 1} \right\}}} ||{\mathcal{L}}oc\left( {F_{i} } \right) - {\mathcal{L}}oc\left( {F_{x} } \right)||$$

2 $${\mathcal{L}}oc\left( F \right) = \left( {u,v} \right)^{T} ,u \in \left[ {0,~W} \right),v \in \left[ {0,H} \right)$$

### Feature matching

In the process of image registration, the moving image needs to find its corresponding positions in the fixed image. This correspondence allows us to transform the positions of feature points ${P}_{M}^{i}$ in the moving image to corresponding positions in the fixed image ${P}_{F}^{i}$ using an Affine transformation matrix $M$. In the fixed image coordinate system, the error between the transformed feature and the corresponding feature is called the projection error, as shown in Eq. 3. *n* is the number of the features in the pair images.

3 $$D_{{proj}} = \sum\limits_{{i = 0}}^{n} {\left\| {P_{F}^{i} - P_{M}^{i} .M} \right\|} ~$$

According to the definition of feature descriptors, although a good descriptor aims to minimize the distance between similar features and increase the distance between dissimilar features, there will always be challenging cases where indistinguishable features are incorrectly matched. According to the statement of Lowe^33^correct matches need to have the closest neighbour significantly closer than the closest incorrect match to achieve reliable matching. We introduce a correlation matrix in the feature-matching method proposed in this work. In this matrix, rows and columns represent indices of features from different modalities. Each entry in the matrix represents the Euclidean distance between features from two distinct modalities, denoted as ${H}_{cor}$. By comparing the top two values ${H}_{cor}$ obtained from sorting the values in each row in ascending order, it is possible to determine whether a particular point represents a good feature match, as shown in Eq. 4.

4 $$\left\{ {\begin{array}{*{20}l} {true~matching,~|\;~H_{{cor\left[ 0 \right]}} \le H_{{cor\left[ 1 \right]}} \;~ \cdot 0.75} \\ {false~matching,~\;|\;~H_{{cor\left[ 0 \right]}} > H_{{cor\left[ 1 \right]}} \;~ \cdot 0.75} \\ \end{array} } \right.$$

According to Eq. 4, a reliable match should have a much smaller distance to the closest neighbour than to the closest incorrect match. In other words, if the distance between the best match ${(H}_{cor\left[0\right]})\:$and the second-best match ${(H}_{cor\left[1\right]})\:$ is large enough, it indicates that the best match is distinctive and reliable. On the other hand, if the distance between the best match and the second-best match is small, it implies that the best match is ambiguous and prone to error. Therefore, we use a threshold of 0.75 to filter out the false matches, as shown in Eq. 4.

**Fig. 6:**
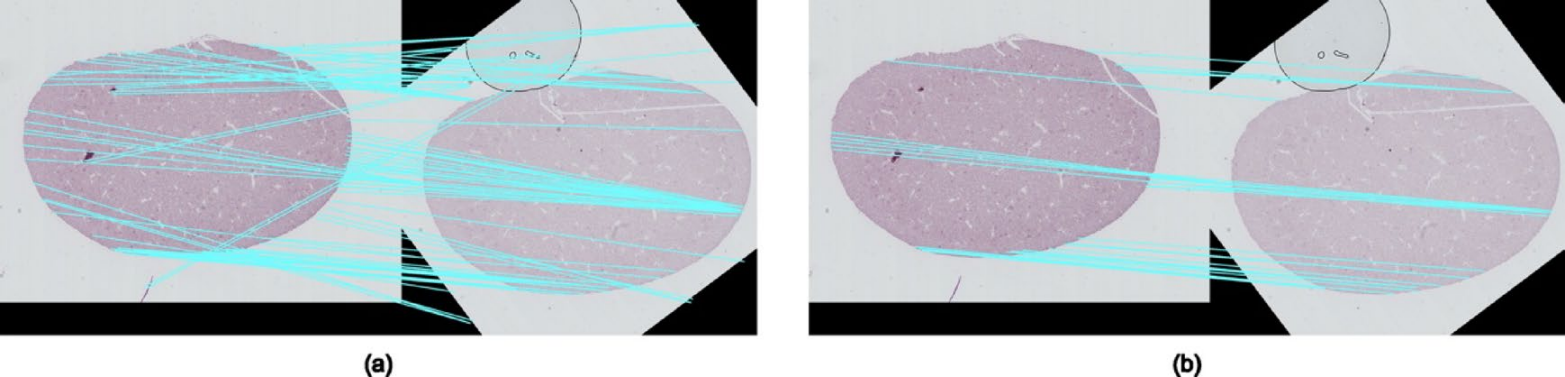
(**a**) Matched features without positional constraints, where features at the bottom of the first image are incorrectly matched to the top of the second image. The blue lines linking falsely matched features create many crossings with correctly matched features. (**b**) Feature matches with positional constraints result in clear and correct alignment.

The feature detection approach in this method focuses on searching for high-frequency areas within the image (Fig. 5). Feature matching is determined by the closest distance between feature descriptions. Then, features from different modalities are matched based on the most similar feature description, which represents the intensity distribution across multiple working window sizes. However, without positional constraints, closest features can be matched across two distinct tissue locations, leading to incorrect matches. For example, observe the initial matching results (straight lines in light blue) in the Fig. 6a. Assuming an affine transformation between the moving and fixed image s, we expect a perspective transformation between matched features on the two planes. Filtering the matches based on this condition significantly improves the matching results, as an example in the Fig. 6b.

Given that neural networks (CNNs), typically require large datasets, our approach avoids these methods in favour of a more resource-efficient solution. This is important in medical tasks and applications where high computational demands can increase costs, potentially limiting access to advanced medical technologies. Inspired by pyramidal analysis, we designed a novel, unsupervised feature extraction method. This method identifies features with high local intensity variation and handles the noise and brightness differences between modalities. This design improves feature matching in multi-modal medical images without relying on the computationally intensive methods often associated with deep learning. Hence this approach provides a viable solution for scenarios where deep learning may not be feasible due to data or resource limitations.

### **Implementation**

The proposed image registration pipeline for the Intensity-based approach has been implemented and executed using the Elastix^52^. The feature-based method runs on a Docker-based Linux system hosted on a MacBook Pro (2019). The registration environment is configured with the following resources: CPU model - Intel(R) Core(TM) i7-8569U @ 2.80 GHz with 1 core, and 1 GiB of memory. The registration process takes approximately 2 min and 11 s for the entire samples listed in Table 1 while it takes 4 min for the intensity based image registration.

### **Accuracy evaluation**

#### **Qualitative evaluation of registration**

To conduct a qualitative evaluation of the registration results, we utilized Napari, an open-source visualization tool, to overlay the registered image onto the fixed image. For this overlay, we adjusted the opacity settings to allow the underlying background image to remain visible beneath the upper image layer.

#### **Quantitative evaluation**

To quantitatively assess our registration approach, we employed two widely recognized metrics: Dice Coefficients and the Hausdorff Distance values^53–55^. These metrics play a crucial role in quantifying the spatial overlap and alignment accuracy between the registered and fixed images. Dice Coefficients, often used in image processing and medical imaging, quantify the similarity or overlap between two sets, in this case, the registered and fixed image regions. It provides a value between 0 and 1, where 1 indicates perfect overlap, and lower values indicate less overlap. The Haimage regionsce values, on the other hand, measure the maximum distance between corresponding points in two sets, capturing the extent of misalignment or mismatch between the registered and fixed images. Smaller Hausdorff Distance values indicate better alignment. By using both of these two metrics, we better assess registration accuracy. The Dice Coefficient evaluates the degree of overlap, ensuring that the registered features align well with the fixed image features, while the Hausdorff Distance value highlights the maximal misalignment. To evaluate these metrics, we identified common features between the fixed image and the registered image, annotating them using polygon selection in Fiji. These annotated features served as the basis for Dice Coefficient and Hausdorff Distance calculations.

## Results and discussion

### **Intensity-based image registration**

#### **Optimization of image registration parameters**

**Fig. 7:**
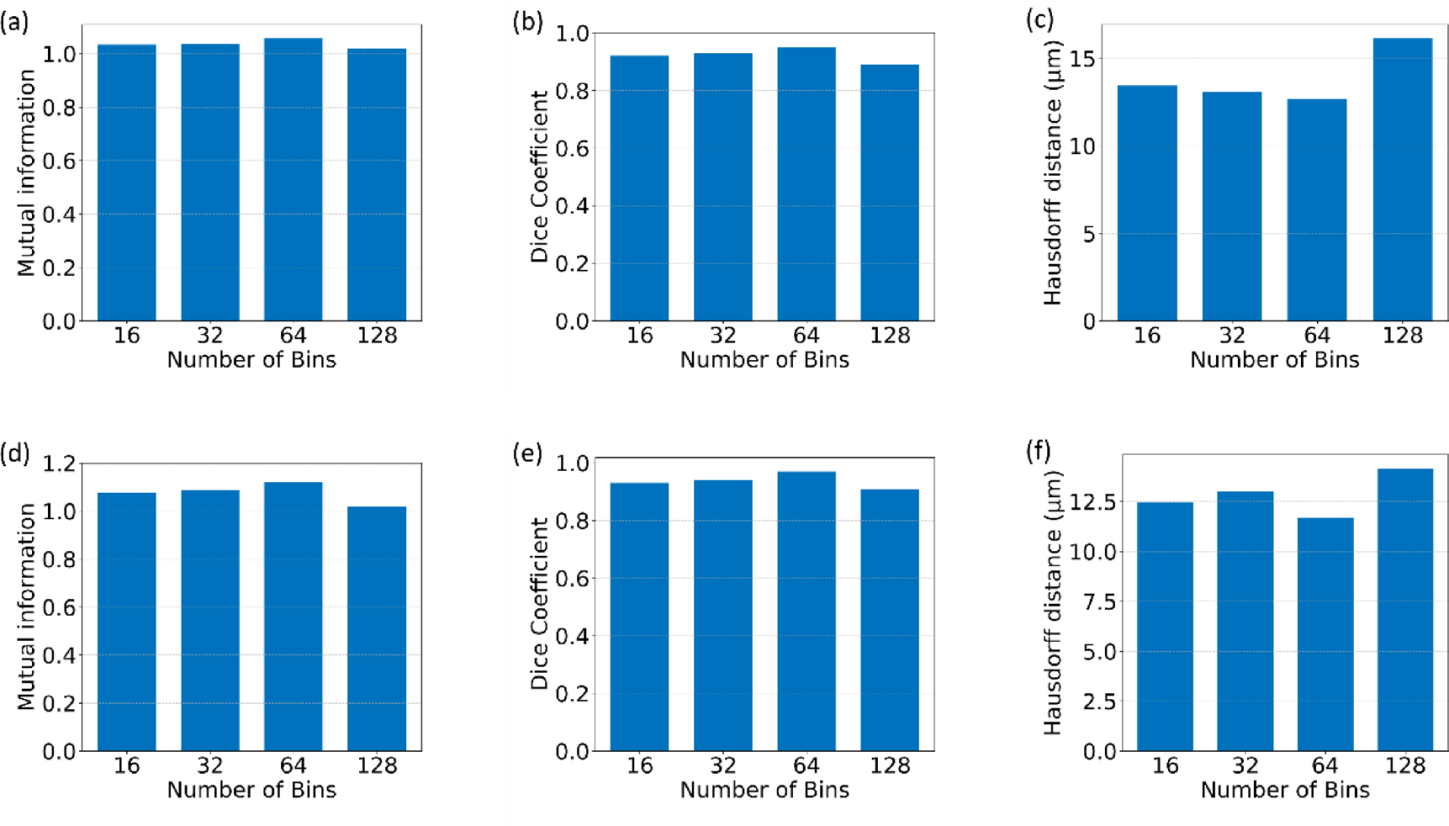
Parameter optimization for intensity-based registration: (**a**–**c**) Results for the COAD_05 sample from the ANHIR dataset, showing mutual information (**a**), Dice Coefficients (**b**), and Hausdorff Distance values (**c**) as functions of the number of bins. (**d**–**f**) Results for the MALDI-MSI samples, showing Mutual Information (**d**), Dice Coefficient (**e**), and Hausdorff Distance (**f**) as functions of the number of bins. Optimal performance is achieved at 64 bins for both samples.

**Fig. 8:**
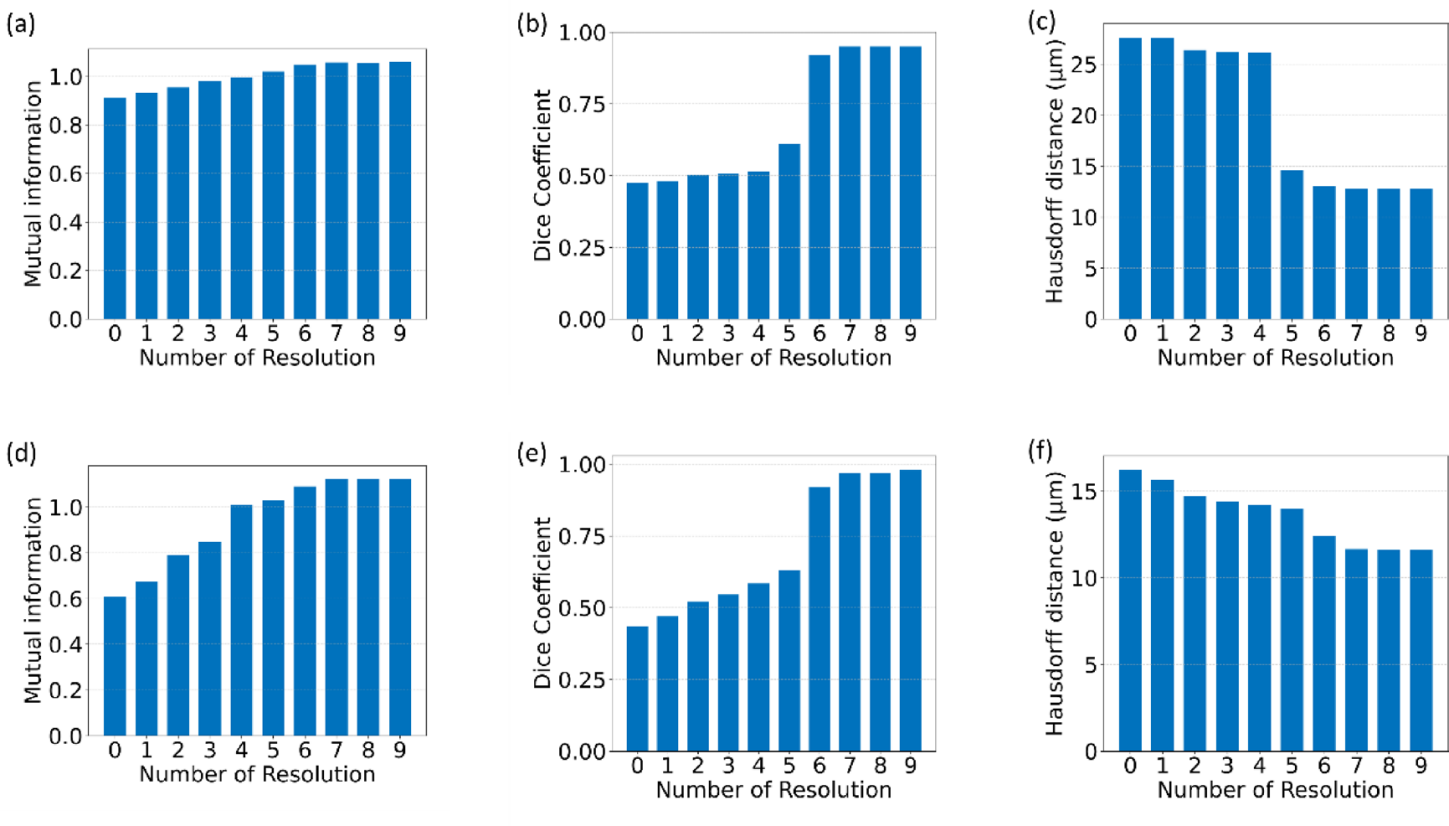
Optimization of parameters for intensity-based registration: (**a**–**c**) Analysis for the COAD_05 sample from the ANHIR dataset, illustrating mutual information (**a**), Dice Coefficient (**b**), and Hausdorff Distance (**c**) as they vary with the number of resolutions. (**d**–**f**) Analysis for the MALDI-MSI samples, depicting Mutual Information (**d**), Dice Coefficient (**e**), and Hausdorff Distance (**f**) based on the number of resolutions. The results indicate that the optimal performance for both samples is achieved with 7 resolutions.

To ensure a robust comparison between our proposed feature-based model and the intensity image registration model, we first optimized the parameters of the intensity registration pipeline. The precision and accuracy of the registration process hinge on several crucial parameters, among which the Number of Bins and the Number of Resolutions play pivotal roles. The Number of Bins parameter influences the histogram used in mutual information computation. A higher number can capture more detailed intensity relationships but may also introduce noise in the Mutual Information computation. The Number of Resolutions parameter, on the other hand, controls the multi-resolution scheme of the registration, where a higher number means more levels in the image pyramid. To identify the optimal values for these parameters, we conducted an extensive analysis where we varied these parameters and evaluated their impact on registration accuracy. We performed image registration for different values of these parameters and calculated the corresponding accuracy metrics. Figure 7 presents the accuracy metrics for varying Number of Bins, while Fig. 8 illustrates the impact of changing the Number of Resolutions on registration accuracy. This optimization analysis was conducted for Affine registration; however, Euler and Similarity registration exhibited similar trends. As shown in Figs. 7 and 8, the Mutual Information and Dice coefficient are highest, and the Hausdorff Distance value is lowest for a Number of Bins equal to 64 and a Number of Resolutions equal to 7. These parameter values provided the highest accuracy and were therefore selected for the intensity-based image registration between different modality pairs. Following this optimization, we compared our proposed model with the optimized intensity registration model to assess relative performance.

#### **Image registration for ANHIR grand challenge datasets**

**Fig. 9:**
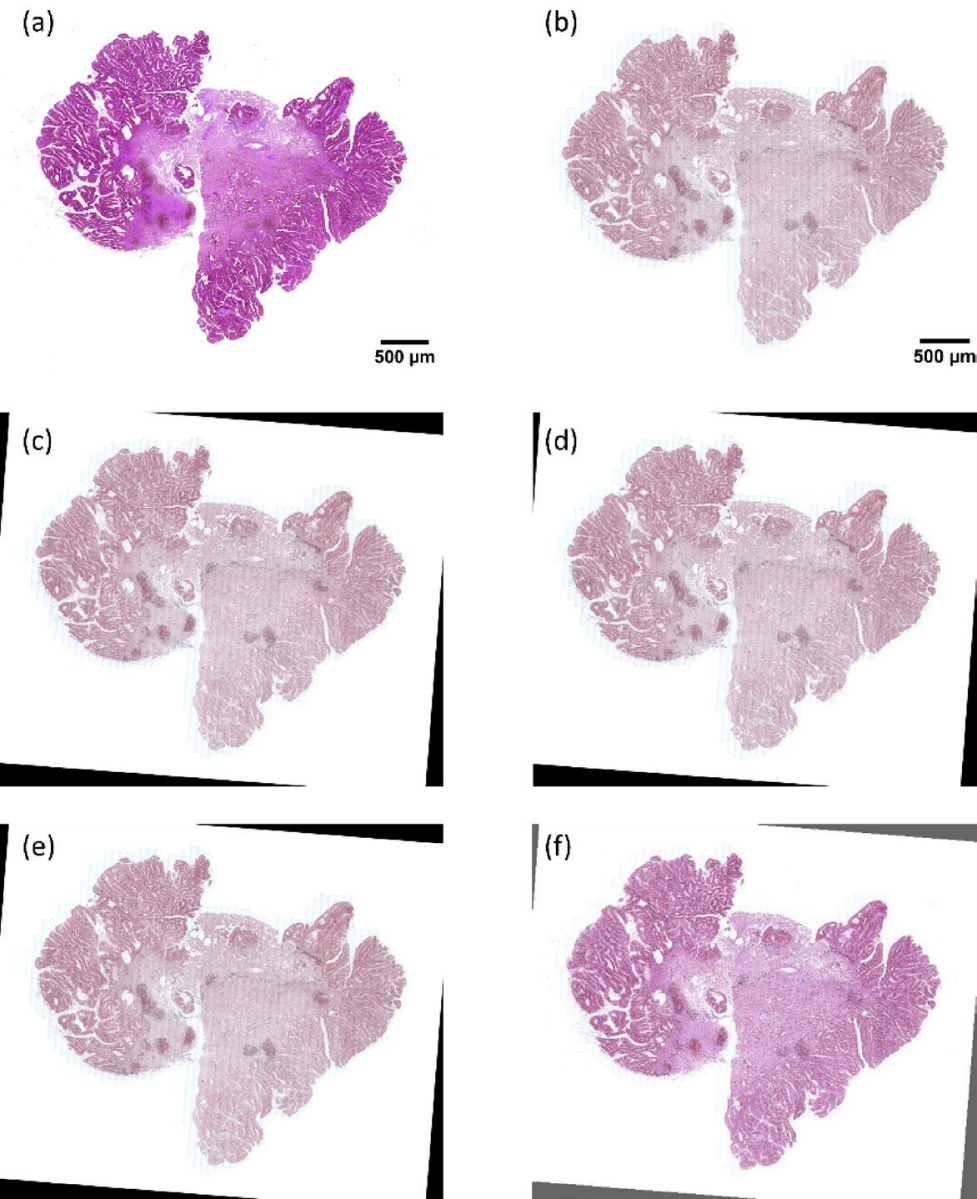
Results of intensity-based image registration for the COAD_05 sample: (**a**) The fixed image serving as the reference, (**b**) The moving image to be aligned with the fixed image, (**c**) Registration outcome using the Euler method, (**d**) Registration outcome using the Similarity method, (**e**) Registration outcome using the Affine method, and (**f**) Overlay of the Affine registration result on the fixed image, highlighting the alignment precision.

We performed image registration on the ANHIR Grand Challenge datasets using elastic models with three transformation approaches: Euler, Similarity, and Affine. Using optimized parameters from our previous analysis, we evaluated the registration accuracy across different tissue samples. Figure 9 demonstrate the registration results for representative cases, while additional examples are provided in the supplementary materials.

Figure 9, along with the figures in the supplementary file, illustrate the results of the image registration experiments using Euler, similarity, and affine transformations within the elastic model framework. All three approaches provided good results, successfully aligning the images with high accuracy across the majority of cases. However, upon closer inspection, it is evident that the affine registration method slightly outperforms the Euler and similarity approaches. Given these results, we chose to use affine registration as a baseline for comparison with our new approach. This allowed us to assess the improvements and advantages offered by our novel method in achieving even more accurate image alignments, particularly in more challenging cases involving complex tissue structures and varying imaging modalities.

#### **Image registration for LA-ICP-MSI with MALDI-MSI**

To evaluate the robustness and versatility of the elastic model and our newly developed approach, we tested both methods on different imaging modalities. Specifically, we focused on the registration of LA-ICP-MSI and MALDI-MSI datasets, which capture distinct but complementary molecular information from samples. In this case, we used MALDI-MSI as the fixed image modality and LA-ICP-MSI as the moving image modality. LA-ICP-MS measures elements such as Fe, Zn, and Au, which are critical for understanding the distribution of these elements within the sample, while MALDI-MSI provide information about lipids and metabolites. Our goal was to assess the ability of both methods to handle the complexity of multimodal registration, where the characteristics of the images differ significantly due to the underlying imaging techniques. Registering these datasets presents distinct challenges in terms of intensity, resolution, the distinct amount of noise associated with each measurement, and the type of information captured, making them an ideal test case for evaluating the performance of our approach.

**Fig. 10:**
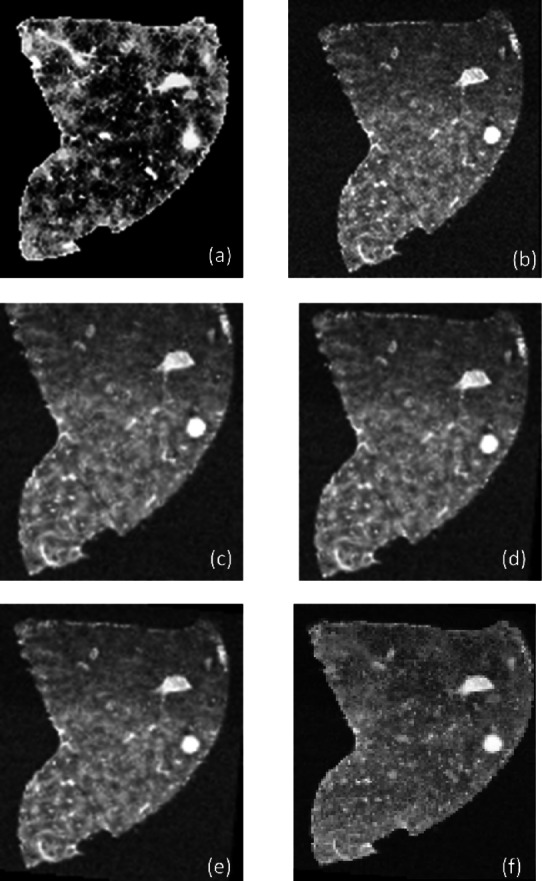
Image registration results for MALDI-MSI and LA-ICP-MSI. (**a**) MALDI-MSI image (fixed modality), (**b**) LA-ICP-MSI image of Fe (moving modality), (**c**) registration result using Euler transformation, (**d**) registration result using similarity transformation, (**e**) registration result using affine transformation, and (**f**) overlay of the Affine-registered LA-ICP-MSI image over the MALDI-MSI image, demonstrating the alignment accuracy. We used t-SNE image for MALDI for image registration.

Our cross-modality registration analysis in Fig. 10 revealed interesting contrasts with the ANHIR dataset results. Unlike the ANHIR findings, Euler transformation showed notably lower accuracy when registering LA-ICP-MSI with MALDI-MSI data. This discrepancy likely stems from the inherent differences between mass spectrometry imaging modalities, which require more complex transformations to achieve proper alignment. Both affine and similarity transformations successfully registered the LA-ICP-MSI Fe distribution map with the MALDI-MSI (obtained by t-SNE) fixed image, demonstrating comparable performance levels. This suggests that these transformation models effectively capture the spatial relationships between molecular and elemental distributions across these imaging techniques. Based on the comparable performance between affine and similarity transformations, we selected the affine registration results as our baseline for comparison with our proposed feature-based approach. This choice was motivated by affine transformation’s ability to handle both rotation and scaling while maintaining computational efficiency.

### **Feature-based image registration**

#### **Image registration for ANHIR grand challenge**

**Fig. 11:**
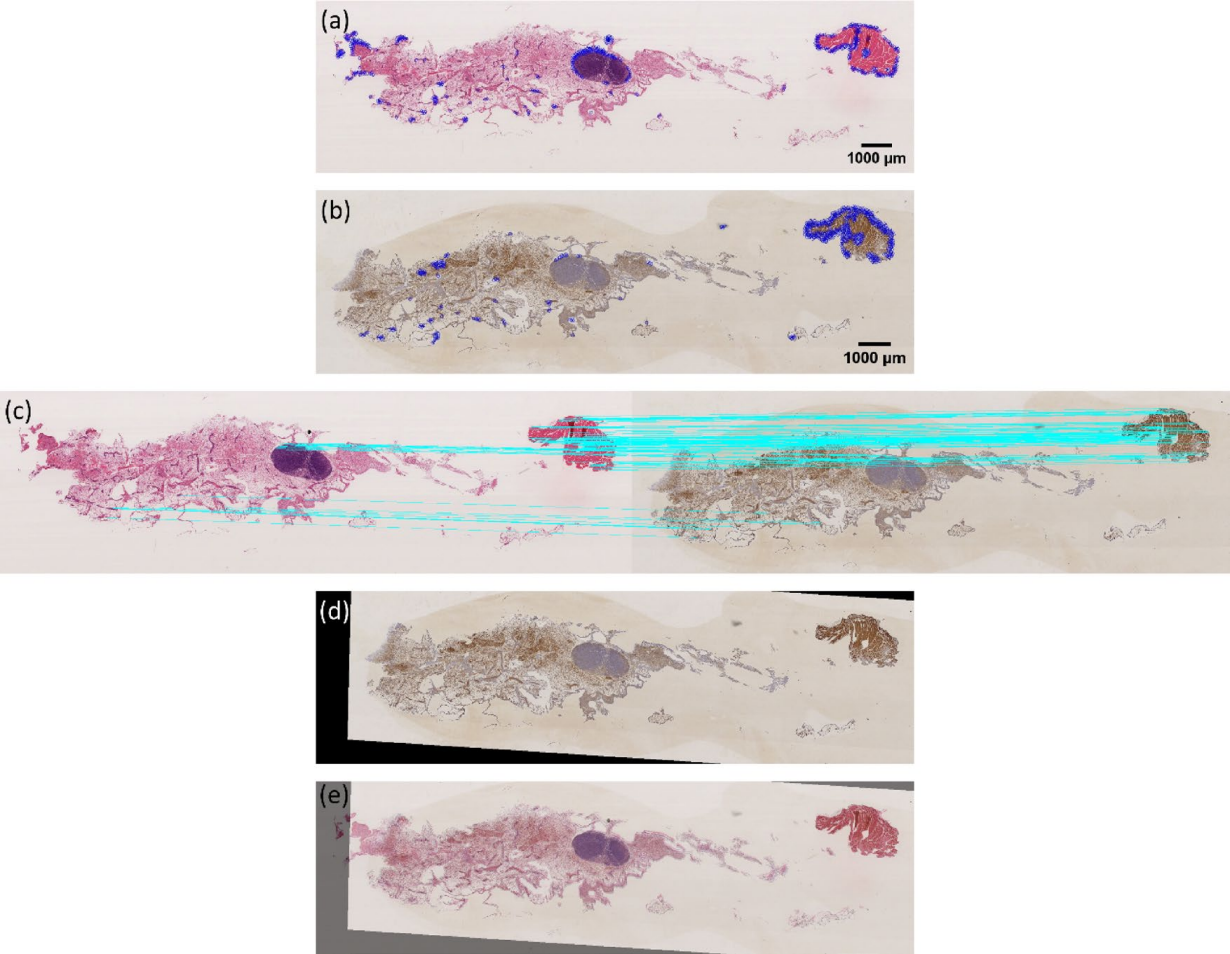
Results of feature-based image registration using our new approach on the Mammary-gland_1 sample. (**a**) Detected key points in the fixed image, (**b**) detected key points in the moving image, (**c**) feature correspondence between the two modalities, (**d**) image registration outcome using our approach, and (**e**) overlay of the registered image on the fixed image, illustrating the quality of the alignment.

**Fig. 12:**
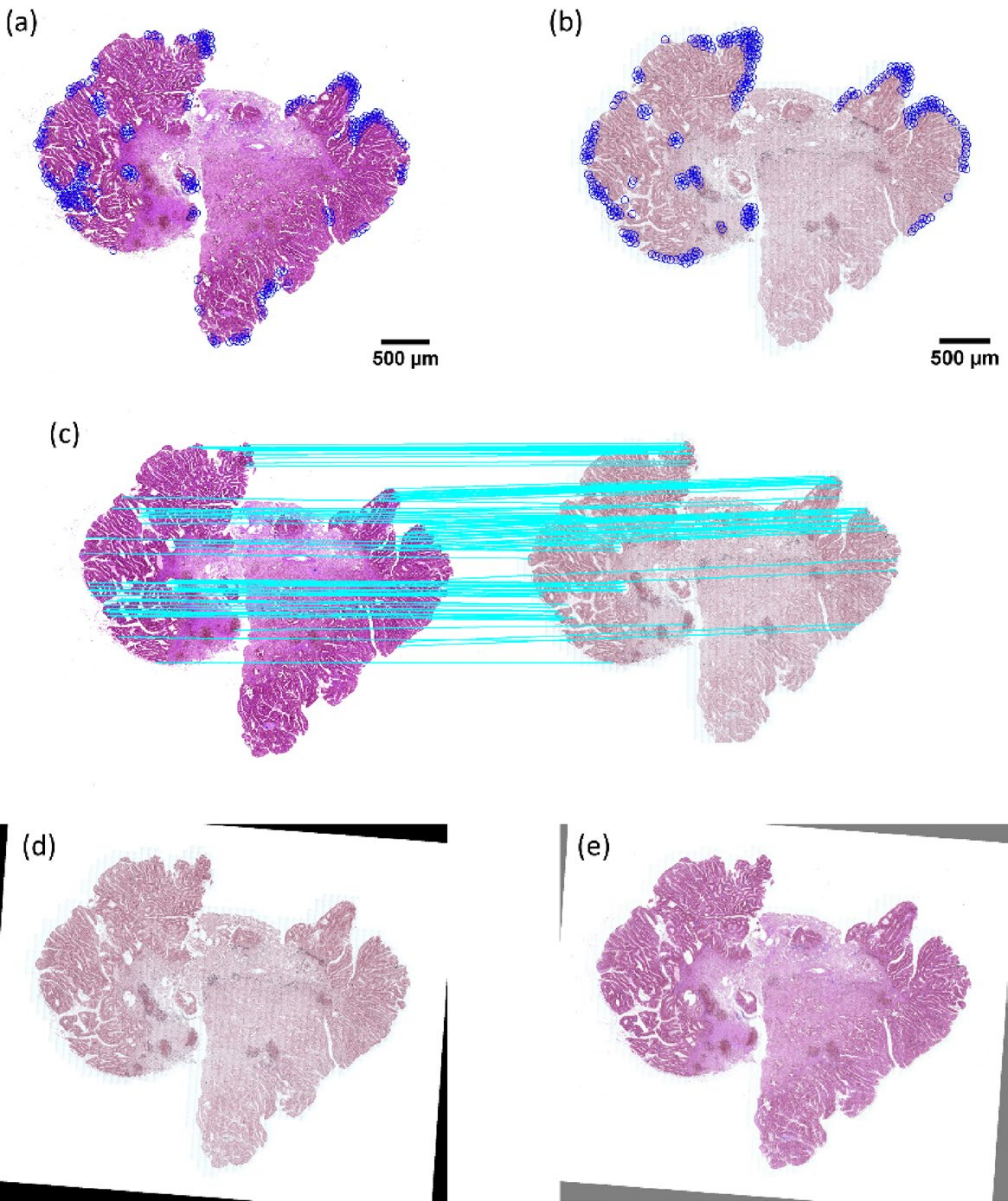
Results of feature-based image registration using our new approach on the COAD_05 sample. (**a**) Key points detected in the fixed image, (**b**) key points detected in the moving image, (**c**) feature mapping between the two modalities, (**d**) image registration result using our approach, and (**e**) overlay of the registered image over the fixed image, demonstrating the accuracy of the alignment.

We tested our newly proposed feature-based image registration method on the ANHIR Grand Challenge dataset to evaluate its performance across different imaging scenarios. The results for two representative samples, COAD_05 and Mammary-gland_1, are presented in Figs. 11 and 12, respectively. For other samples the results are presented in Supplementary Information.

The results in Figs. 11 and 12 demonstrate the effectiveness of our approach in accurately aligning images from distinct modalities, highlighting the robustness of the feature detection and matching process. For the COAD_05 sample, the key points detected in both the fixed and moving images were successfully mapped. The two ends of each blue line indicate visually similar textures and the same position on the tissue, resulting in precise registration outcomes. Similarly, for the Mammary-gland_1 sample, our method showed strong alignment capabilities. The feature matches in (c) are correct and consistent, making it difficult to discern differences in the tissue outline in the overlay image. This indicates the method’s adaptability across diverse tissue types and imaging characteristics. Additional registration results for more samples from the ANHIR Grand Challenge dataset are provided in the supplementary file, offering further validation of our method’s performance. These results collectively support the efficacy of our new feature-based image registration approach, suggesting it can effectively handle complex multimodal image registrations across different sample types.

#### **Image Registration for LA-ICP-MSI with MALDI-MSI**

**Fig. 13:**
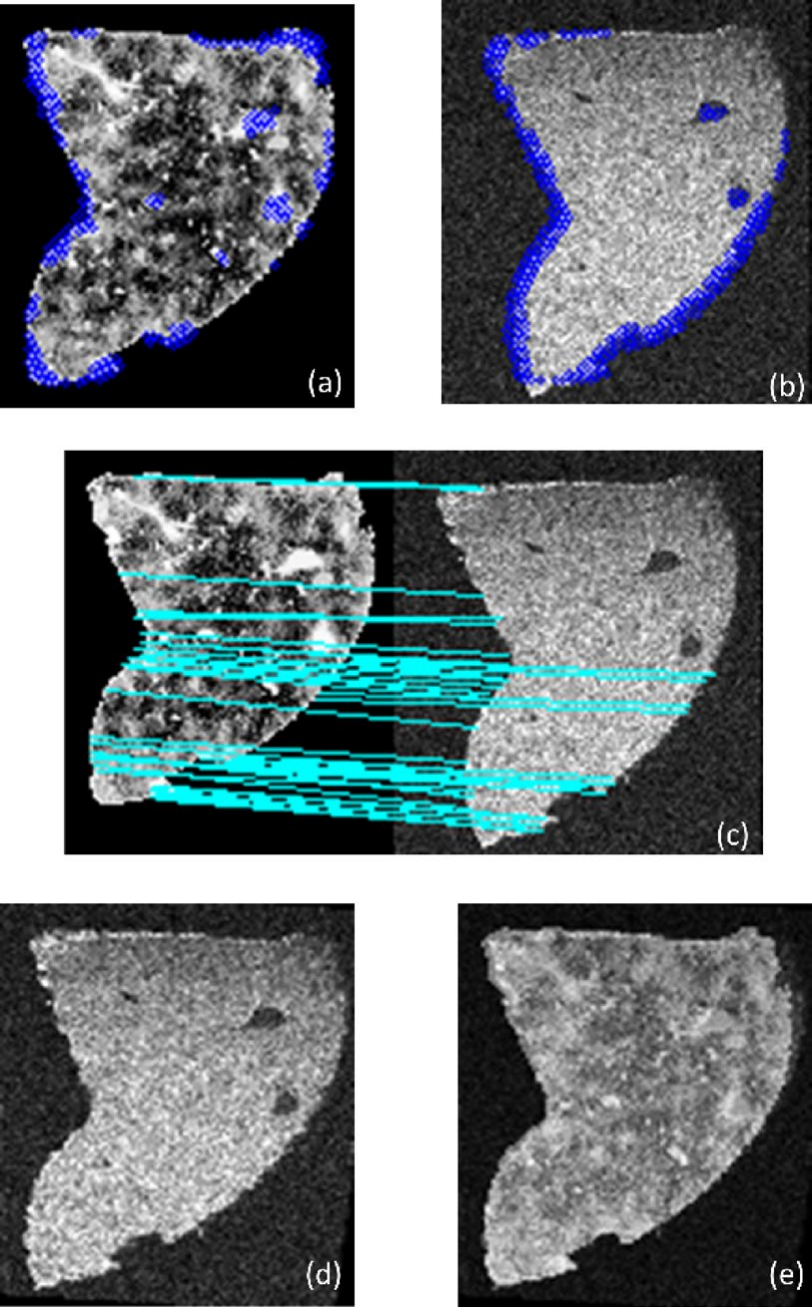
Results of feature-based image registration using our new approach on LA-ICP-MSI (Fe) and MALDI-MSI. (**a**) Key points detected in the MALDI-MSI (fixed modality), (**b**) key points detected in the LA-ICP-MSI (Fe, moving modality), (**c**) feature mapping between the two modalities, (**d**) image registration result using our approach, and (**e**) overlay of the registered LA-ICP-MSI (Fe) image over the MALDI-MSI image, demonstrating the accuracy of the alignment.

To assess the effectiveness of our new approach across different imaging modalities, we also tested it on the LA-ICP-MSI and MALDI-MSI datasets. In this case, LA-ICP-MSI was used as the moving image, while MALDI-MSI served as the fixed image. This multimodal registration task involved aligning elemental distributions from LA-ICP-MSI with molecular profiles from MALDI-MSI (obtained by t-SNE). The results for Fe in the LA-ICP-MSI dataset are presented in Fig. 13.

**Fig. 14:**
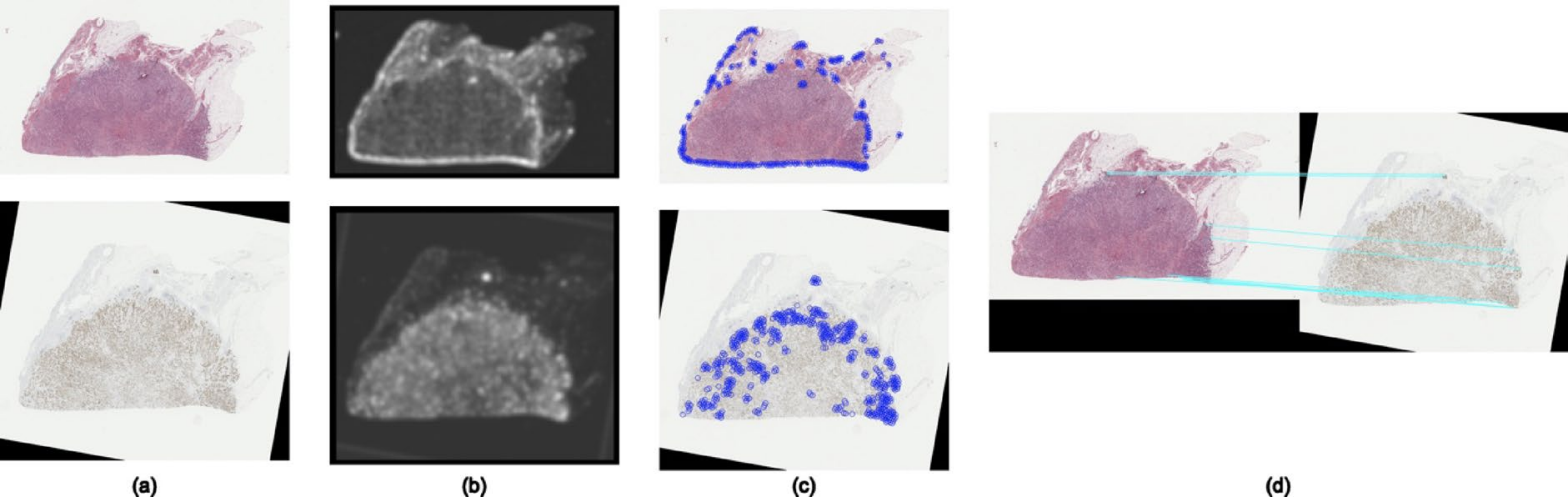
Results of feature-based image registration on sample Breast_4. (**a**) original images, (**b**) feature magnitude maps, (**c**) detected features, (**d**) feature matching results.

The results presented in Fig. 13 demonstrate the effectiveness of our new feature-based image registration approach for aligning LA-ICP-MSI (Fe) and MALDI-MSI images. Despite these differences, our approach efficiently matched and transformed the key points across the two modalities, ensuring accurate spatial alignment. The feature mapping between the two modalities shows the robustness of our key point detection and matching process, which is crucial for establishing a reliable transformation between the images. Similar to the result in Fig. 14, this registration result can be mapped identically to the fixed image, further highlights how well the LA-ICP-MSI image (Fe) aligns with the MALDI-MSI image after applying our approach. In addition, the overlay (panel e) provides a visual confirmation of the accuracy of the alignment, with the registered LA-ICP-MSI image perfectly overlaying onto the MALDI-MSI image. This overlay illustrates how our method can effectively handle different image modalities and capture the spatial correspondence between them, even when the modalities differ significantly in terms of information type. These results underscore the potential of our approach for multimodal image registration tasks, providing a powerful tool for studies that require precise alignment of complex and heterogeneous imaging data. For further validation, the results for Au and Zn from the LA-ICP-MSI dataset are provided in the supplementary file, showcasing that our method consistently performs well across multiple elemental distributions. These findings underscore the versatility and robustness of our approach for multimodal image registration, offering an effective solution for studies that require precise alignment of complex, heterogeneous imaging data.

## **Limitations of feature matching**

The feature detection method proposed in this project selects features based on the intensity variation within a working window. It performs well in identifying regions with high intensity changes, as demonstrated by the results above. However, the feature selection process relies on the feature magnitude map, which is a global representation of all features in the entire image, where all features are ranked by the same magnitude scale. For samples with inconsistent intensity across different regions, the feature magnitude maps can vary significantly. In the sample shown in Fig. 14a, the upper-left image shows the left edge of the tissue with a consistent intensity compared to the background. In contrast, the lower-left image exhibits a clear intensity boundary within the tissue and a distinctive black dot in the top-middle region of the tissue. This leads to two different feature distributions, as shown in Fig. 14c. As a result, the filtered feature pairs in Fig. 14d are fewer and less accurate, as illustrated by the incorrect feature pair (light blue line) at the top.

### **Quantitative evaluation**

#### Comparison of intensity-based registration methods: euler, similarity, and affine

**Fig. 15:**
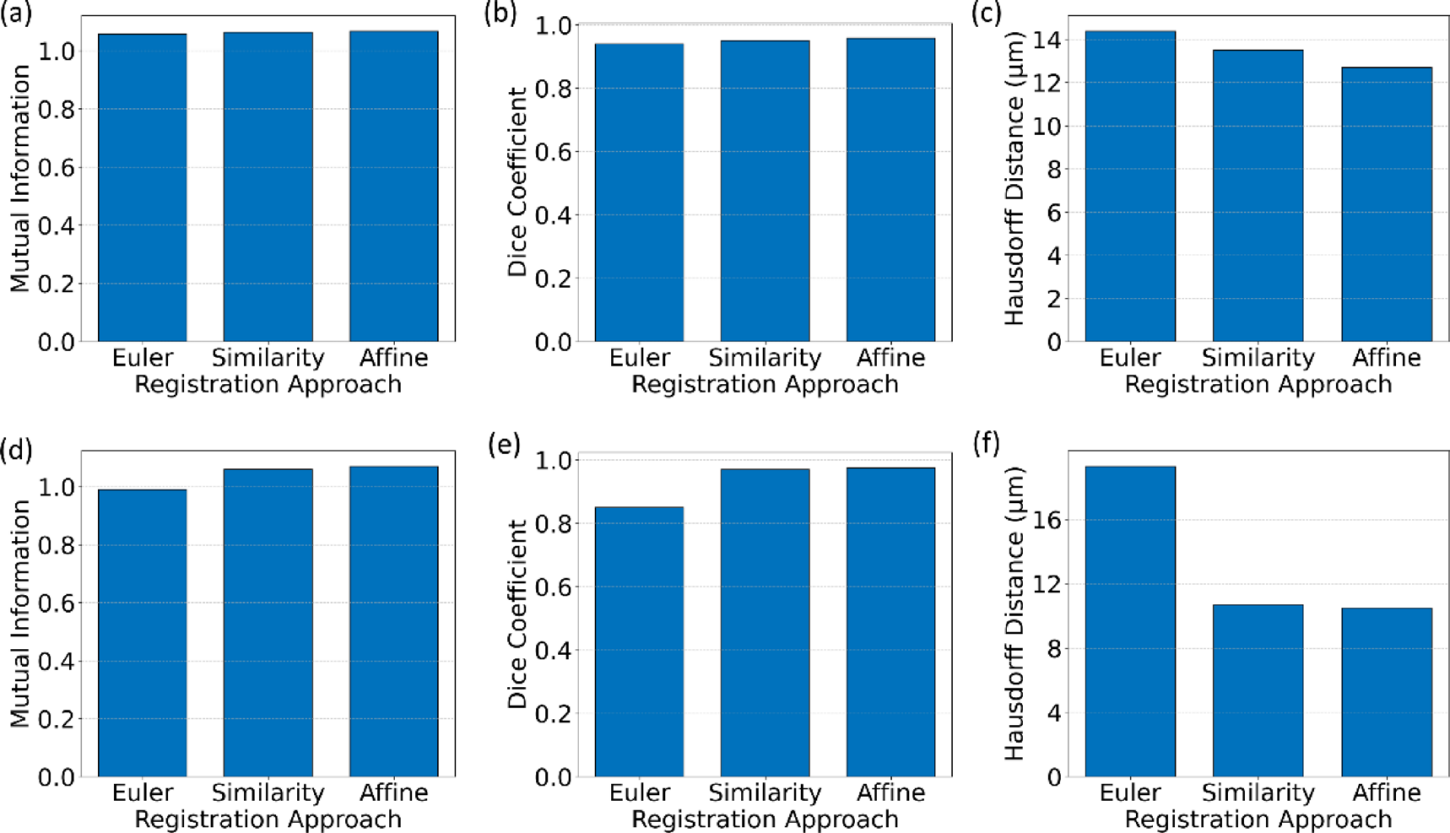
Quantitative evaluation of intensity-based registration methods: (**a**–**c**) Results for the COAD_05 sample from the ANHIR Grand Challenge dataset, showing Mutual Information (**a**), Dice Coefficient (**b**), and Hausdorff Distance (**c**); (**d**–**f**) Results for the MALDI-MSI with LA-ICP-MS sample, showing Mutual Information (**d**), Dice Coefficient (**e**), and Hausdorff Distance (**f**). These metrics provide a comprehensive assessment of registration accuracy across different methods and datasets.

In this analysis, we evaluate the performance of the Euler, Similarity, and Affine registration methods on the ANHIR Grand Challenge datasets (CODA_05) and MALDI-MSI with LA-ICP-MS. To assess registration accuracy, we employed the Dice Coefficient and Hausdorff Distance evaluations. To obtain these metrics, we identified common features between the fixed and registered images, annotating them using polygon selection in Fiji. The results, presented in Fig. 15.

The results of the quantitative evaluation of the intensity-based method in Fig. 15 reveal distinct trends in the performance of Euler, Similarity, and Affine registration methods across two datasets: the COAD_05 sample from the ANHIR Grand Challenge and the MALDI-MSI with LA-ICP-MS sample. For the COAD_05 sample, all three approaches demonstrated high accuracy. The Affine method outperformed the others, achieving the highest Dice Coefficient (0.958), Mutual Information (MI) value (1.067), and the lowest Hausdorff Distance (12.7). The Similarity method followed closely, with a Dice Coefficient of 0.95 and a Hausdorff Distance of 13.5. The Euler method, while effective, lagged slightly behind with a Dice Coefficient of 0.94 and a Hausdorff Distance of 14.38, reflecting its limitations in handling complex transformations. Similar trends were observed for other samples from the ANHIR Grand Challenge, where Affine consistently outperformed the other methods, followed by Similarity. Euler often demonstrated adequate but relatively weaker performance, particularly for datasets requiring more intricate alignments. For the MALDI-MSI with LA-ICP-MS sample, the performance trends were similar, but the Euler method’s results were noticeably lower compared to its performance on the COAD_05 sample. The Affine method once again achieved the best performance with a Dice Coefficient of 0.975, an MI of 1.07, and the lowest Hausdorff Distance of 10.5. The Similarity method closely followed with a Dice Coefficient of 0.97 and a Hausdorff Distance of 10.7. However, the Euler method’s Dice Coefficient dropped to 0.85, and its Hausdorff Distance increased to 19.3, indicating its difficulty in registering the complex multimodal images of this dataset.

#### **Comparison of feature-based and intensity-based registration approaches**

We compare our new feature-based registration approach with the affine registration method, which is based on intensity, across different imaging modalities. The results are presented in Tables 2 and 3.

**Table 2:** Comparison of affine and Feature-Based registration approaches for the COAD_05 sample from the ANHIR grand challenge dataset, showing performance metrics: mutual information, Dice Coefficient, and Hausdorff Distance (µm).

Registration approach	COAD_05 sample		
	Mutual information	Dice coefficient	Hausdorff distance (µm)
Affine	1.06	0.95	12.5
Feature-based	1.04	0.95	12.7

**Table 3:** Performance metrics for affine and Feature-Based registration methods on the MALDI-MSI with LA-ICP-MS sample, including mutual information, Dice Coefficient, and Hausdorff Distance (µm).

Registration approach	MALDI-MSI with LA-ICP-MS		
	Mutual information	Dice coefficient	Hausdorff distance (µm)
Affine	1.07	0.97	10.7
Feature-based	1.05	0.97	10.6

The results presented in Tables 2 and 3 demonstrate that our new feature-based registration approach performs comparably to the affine registration method across two distinct imaging modalities: the COAD_05 sample from the ANHIR Grand Challenge dataset and the MALDI-MSI with LA-ICP-MS sample. For the COAD_05 sample, both registration approaches achieved nearly identical Dice Coefficients of 0.95, indicating a high degree of overlap between the fixed and registered images. The Mutual Information values for both methods are also very similar, with the affine approach yielding a value of 1.06 and the feature-based method producing a value of 1.04. The Hausdorff Distance is slightly better for the affine method at 12.5 μm compared to the feature-based approach at 12.7 μm, though this difference is marginal and still represents a satisfactory registration result for both techniques. Similarly, for the MALDI-MSI with LA-ICP-MS sample, the affine and feature-based approaches show near-identical performance, with Mutual Information values of 1.07 and 1.05, respectively, and Dice Coefficients of 0.97 for both methods. The Hausdorff Distance is almost the same for both approaches at 10.7 μm for affine and 10.6 μm for feature-based registration, again demonstrating very similar alignment accuracy. Overall, these results suggest that the feature-based registration approach provides very satisfactory results, performing almost on par with the affine method. Despite the slight differences in some metrics, the feature-based approach offers competitive registration accuracy and could potentially provide advantages in more complex or varied imaging scenarios with less computational time.

## Conclusion

In this study, we developed and evaluated comprehensive approaches for multimodal image registration, introducing a novel feature-based feature extraction method inspired by SPP-net architecture and compared it to traditional intensity-based techniques. Our analysis encompassed various transformation approaches—Euler, Similarity, and Affine—tested across different imaging modalities including H&E, LA-ICP-MS, and MALDI-MSI datasets. Our proposed feature-based method demonstrated robust performance through its multi-level feature extraction capabilities, achieving comparable results to optimized intensity-based methods with Dice Coefficients of 0.95 for ANHIR dataset samples (COAD_05) and 0.97 for MS imaging data. The intensity-based registration methods, particularly the Affine transformation, showed consistently high accuracy across all test cases, with optimal performance at 64 bins and 7 resolutions. Quantitative evaluation through Mutual Information, Dice Coefficient, and Hausdorff Distance metrics revealed that both approaches achieved high registration accuracy. While the intensity-based Affine method showed marginally better performance in some cases (Mutual Information: 1.06 vs. 1.04 for COAD_05), our feature extraction method offered comparable results with notable advantages in computational efficiency, completing registrations in approximately half the time. These findings demonstrate that our feature-based approach provides a robust alternative to traditional intensity-based methods for multimodal image registration in biomedical applications, with the choice between them potentially depending on specific use case requirements such as computational resources and time constraints. Future work could explore introducing the deep learning architecture and combining these approaches to leverage their complementary strengths in handling complex multimodal registration scenarios.

The corresponding author is responsible for submitting a competing interests statement on behalf of all authors of the paper.

### **Future studies**

Our approach, while currently focused on 2D registration, provides a benchmark that can be adapted and extended to 3D datasets in future studies.

## Electronic supplementary material

Below is the link to the electronic supplementary material.

Supplementary Material 1

## Data Availability

The data and code used in this research will be made publicly available upon acceptance of the manuscript. We will make the code available on our GitHub repository upon acceptance. For any requests regarding the data from this study, please contact the corresponding author.
