# Supplementary material for: Novel feature-based method for multi-modal biomedical image registration compared to intensity-based technique
Source: Sci Rep. 2025 Aug 1;15:28183. doi: 10.1038/s41598-025-12862-2 (PMC12317088; doi:10.1038/s41598-025-12862-2)
Supplement: Supplementary file 1 — Supplementary Material 1 [file 41598_2025_12862_MOESM1_ESM.docx]

**Novel Feature-Based Method for Multi-Modal Biomedical Image Registration Compared to Intensity-Based Technique**

**Mohammad Javad Shojaei**^1^**, Lichen Yang**^2^**, Kazem Shojaei**^3^**, Jeerapat Doungchawee**^4^**, Richard W. Vachet**^4^

^1^Department of Materials, Imperial College London, London, UK

^2^School of Computing, Newcastle University, UK.

^3^Tehran University of Medical Sciences, School of Medicine, Iran

^4^Department of Chemistry, Molecular and Cellular Biology Program, University of Massachusetts Amherst, USA

*Corresponding Author, m.shojaei@imperial.ac.uk

**Intensity-Based Method**

The Intensity-based approach uses information at the pixel level to find a transformation function that makes a moving image spatially aligned with a fixed or target image, as shown in Equation 1 [1].

$\hat{T}_{\gamma}=argminC(T_{\gamma};I_{F},I_{M}) (1)$

where $\hat{T}$ is the estimated transformation that aligns two images $I_{F}$ and $I_{M}$ as the fixed and moving image respectively, the goal of image registration is to find the optimal transformation $T$ that brings the images into a common coordinate system, aligning corresponding features and structures. In other words, image registration looks for a transformation $(T)$that aligns the moving image with the fixed image in the best possible way, and this alignment is achieved by minimizing the cost function. The optimal transformation that achieves this alignment is denoted as $\hat{T}$. The presence of a subscript $\gamma$ denotes that the transform has been parameterized, and we presented the optimised parameters in our proposed pipeline in the appendix. To achieve this, a suitable metric is chosen to measure the similarity or dissimilarity between the images during the registration process. The metric acts as an objective function that guides the registration algorithm towards finding the best alignment. It quantifies how well the transformation parameters align the images and helps the algorithm iteratively refine these parameters until convergence. In this study, Mutual Information was employed as a metric for image registration. Mutual Information is a widely used metric, especially in scenarios involving multi-modal images. It measures the mutual dependence between the intensity distributions of the two images being registered. It is defined as follows [1]:

$$MI(\gamma;I_{F},I_{M})=\sum_{m\in I_{M}} \sum_{m\in I_{F}} \rho(f, m, \gamma){log}_{2}(\frac{\rho(f, m, \gamma)}{\rho_{F}(f)\rho_{M}(m;\gamma)}) (2)$$

where $\rho$ denotes the discrete joint probability. The marginal discrete probabilities of the fixed and moving image s, $\rho_{F}$ and $\rho_{M}$, are obtained by summing $\rho$ over $f$ and $m$, respectively. Each $f$ and $m$ corresponds to a particular feature in the fixed and moving image respectively. In this process, the moving image $I_{M}$ is transformed to match the fixed image $I_{F}$.

By maximizing the mutual information between the images, the registration algorithm effectively identifies the transformation that aligns corresponding structures and features while accounting for potential intensity variations and relationships. To preserve the integrity of the image with no non-linear distortion and prevent any change to its intensity, we opted for Euler, Similarity and Affine registration and avoided non-linear registration.

Euler’s registration is an image registration method involving only rotation and translation transformations, preserving the size and shape of image features as shown in Equation 4 [1].

$$T(x)=R(x-c)+t+c (4)$$

Using the matrix $R$ as a rotation matrix, along with the centre of rotation $c$ and translation $t$. On the other hand, Similarity transformation is defined as [1]:

$$T(x)=sR(x-c)+t+c (5)$$

with $s$ a scalar and $R$ a rotation matrix. Similarity registration is a type of image registration method that involves scaling, rotation, and translation transformations. These transformations mean that the size of the image features can change during the registration process, in addition to their position and orientation.

Affine registration is a transformation that involves rotating, translation, scaling, and shearing to align the imaging datasets. The Affine transformation is defined as [1]:

$$T(x)=A(x-c)+t+c (6)$$

where the matrix $A$ is unrestricted, implying that the image can undergo translation, rotation, scaling, and shearing without limitations which means that the size, shape, and orientation of the image features can change during the registration process. Typically, an iterative optimization approach is used to solve the optimization problem and determine the optimal transformation parameter vectors, *γ*. We utilized the Adaptive Stochastic Gradient Descent as the optimizer [1]:

$\gamma_{k+1}=\gamma_{k}+a_{k}d_{k} (7)$

where $d_{k}$represents the search direction at iteration $k$, while $a_{k}$ is a scalar gain factor that governs the step size along the same search direction. For image registration, we employed different registration approaches. To further understand the performance of different image registration approaches, we applied the same transformation function from each approach to a mesh image, as depicted in Figure S1.


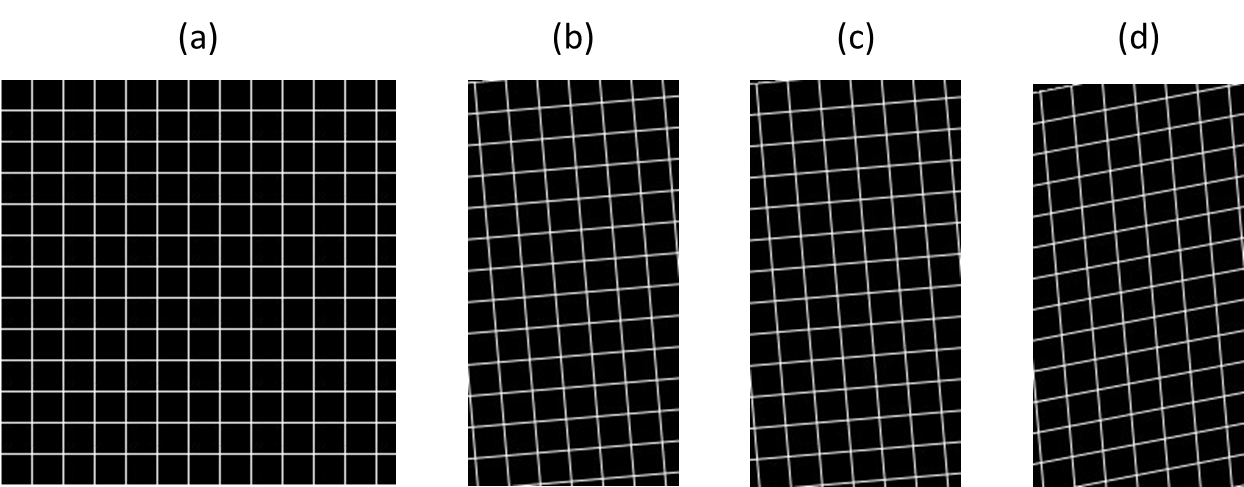


Figure S1. Visualization of transformation effects on a mesh image for different registration approaches. (a) shows the original mesh image. (b), (c), and (d) show the results of image registration using Euler, Similarity, and Affine registration approaches, respectively.

The results reveal distinct characteristics of each method. The Euler transformation performs only rotation and translation, maintaining the square shape of the mesh. The Similarity transformation adds scaling to rotation and translation, but it also preserves the square shape. In contrast, the Affine transformation introduces shearing in addition to scaling, rotation, and translation, altering the square mesh into a diamond shape. These visualizations provide insights into how each method manipulates images during the registration process.


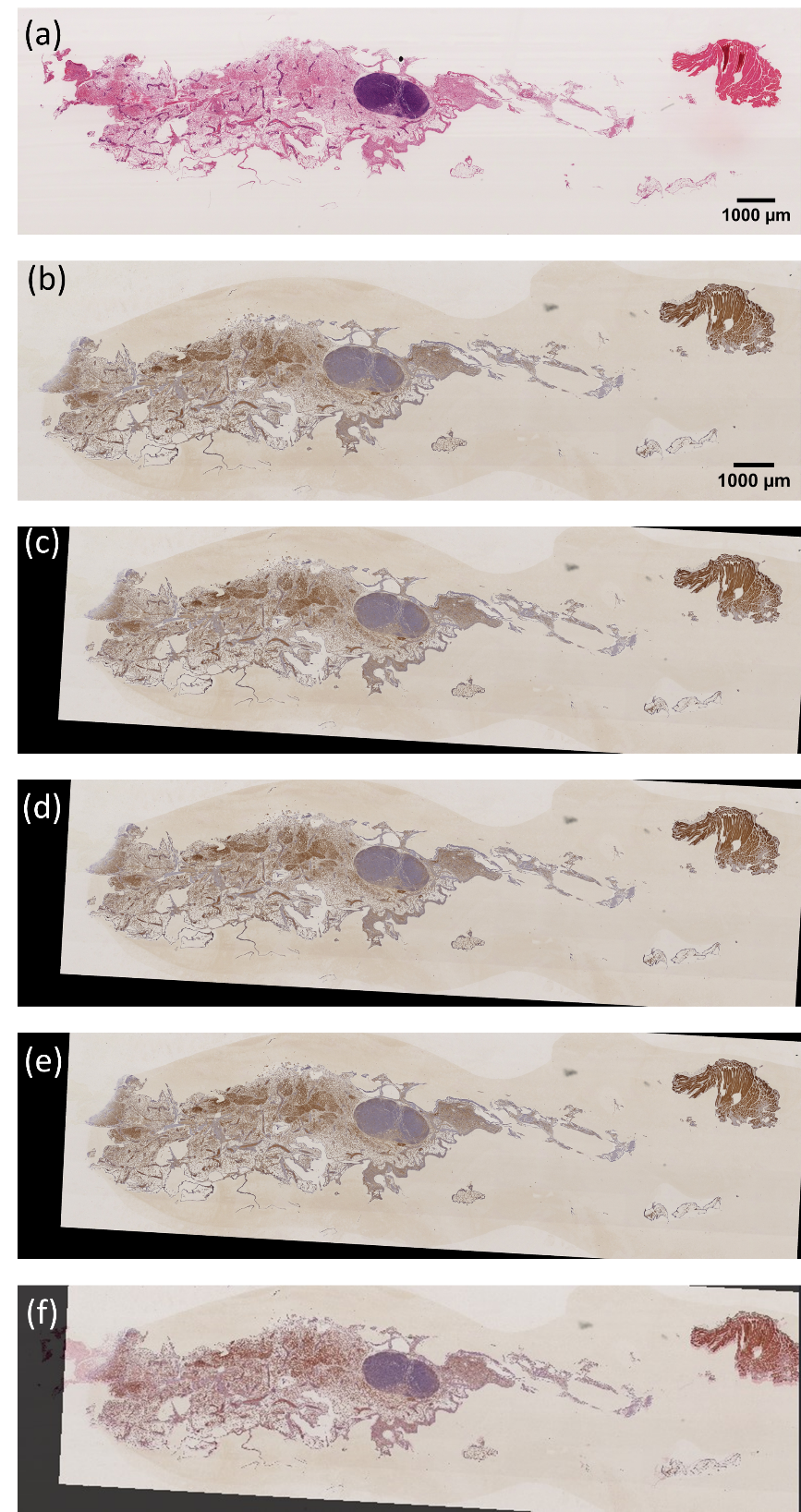


Figure S2. Intensity-based image registration results for the Mammary-gland_1 sample from the ANHIR dataset: (a) fixed image used as the reference for registration, (b) moving image to be aligned with the fixed image , (c) Result of the Euler method, (d) Similarity method, (e) Affine method, and (f) Overlay of the Affine method result on the fixed image, demonstrating alignment accuracy.


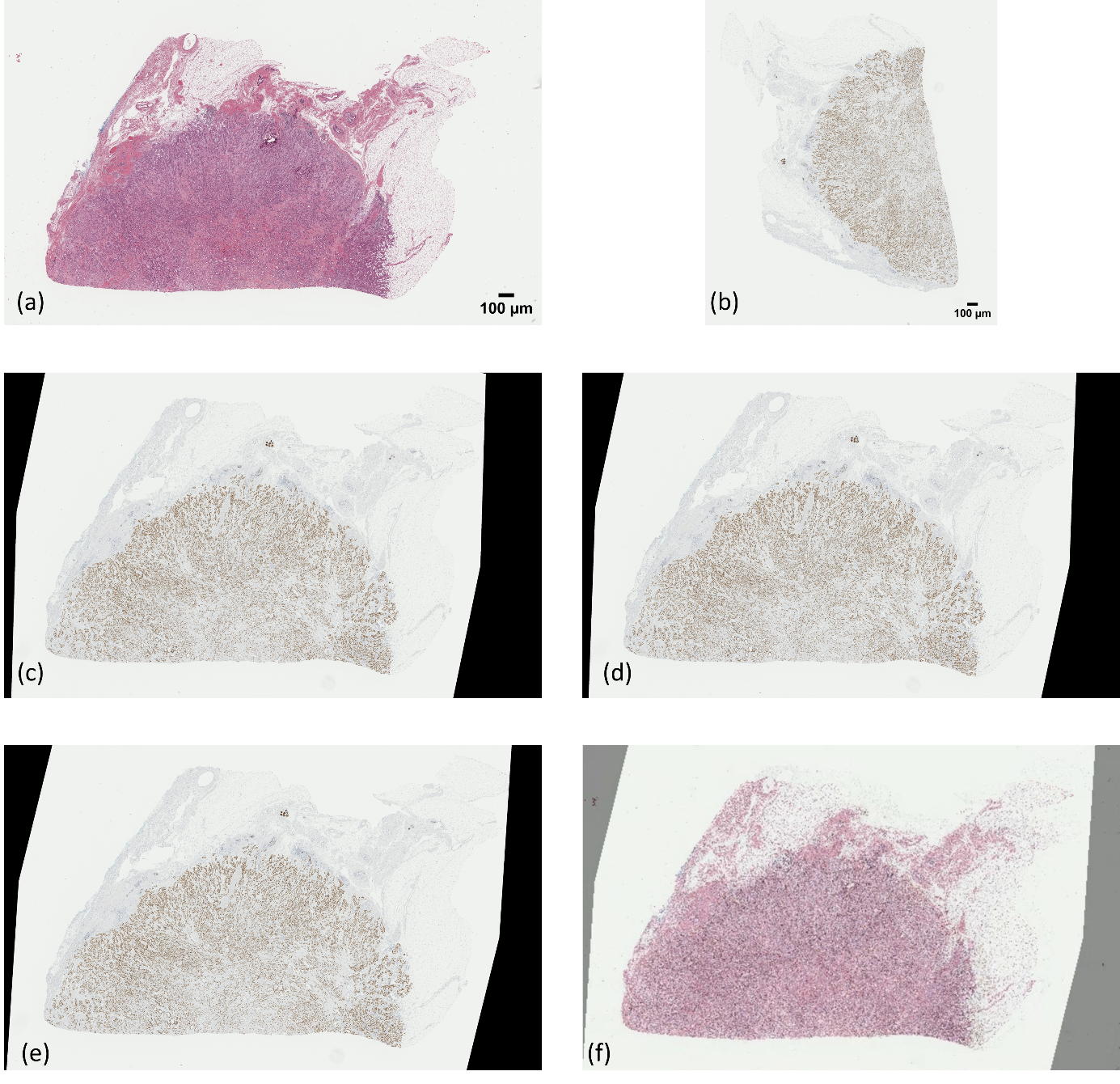


Figure S3. Intensity-based image registration results for the Mammary-gland_1 sample from the ANHIR dataset: (a) Fixed image used as the reference for registration, (b) Moving image to be aligned with the fixed image, (c) Result of the Euler method, (d) Similarity method, (e) Affine method, and (f) Overlay of the Affine method result on the fixed image, demonstrating alignment accuracy.


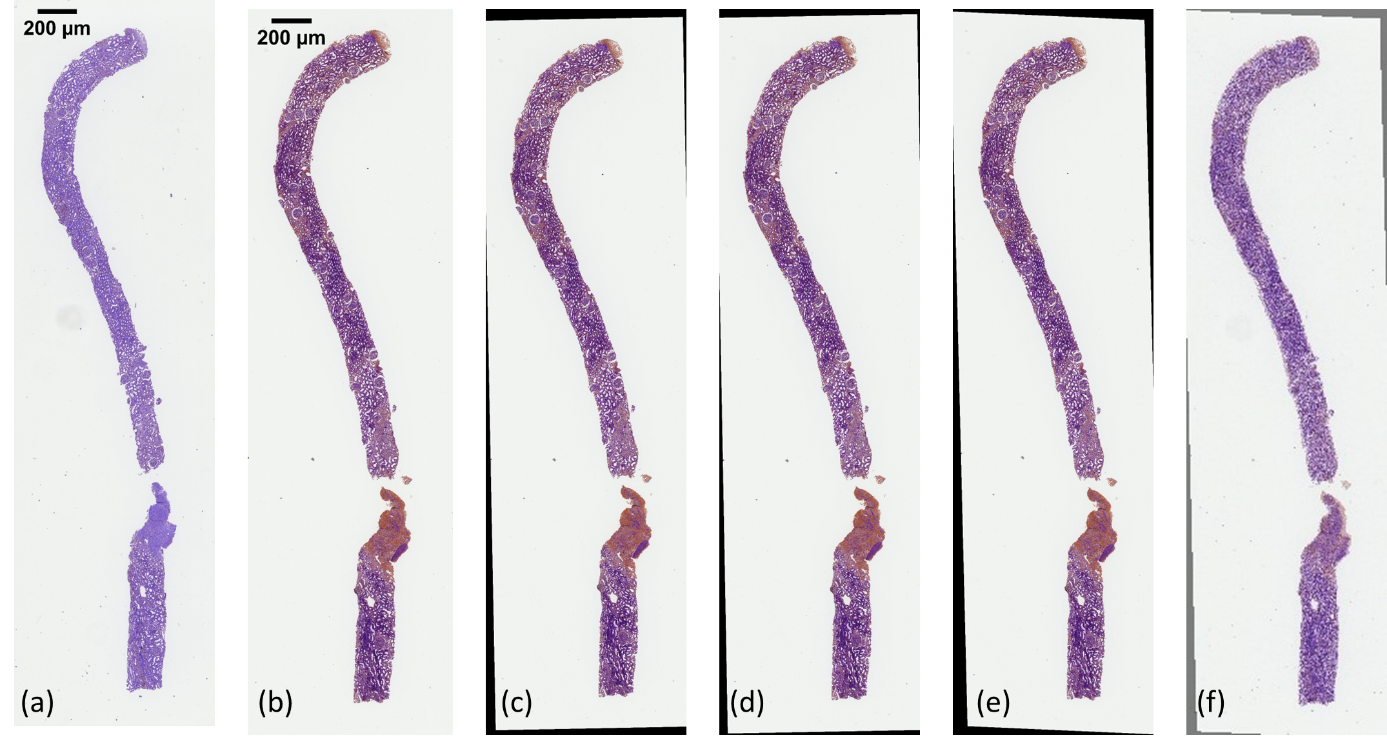


Figure S4. Intensity-based image registration results for the Mammary-gland_1 sample from the ANHIR dataset: (a) Fixed image used as the reference for registration, (b) Moving image to be aligned with the fixed image, (c) Result of the Euler method, (d) Similarity method, (e) Affine method, and (f) Overlay of the Affine method result on the fixed image, demonstrating alignment accuracy.


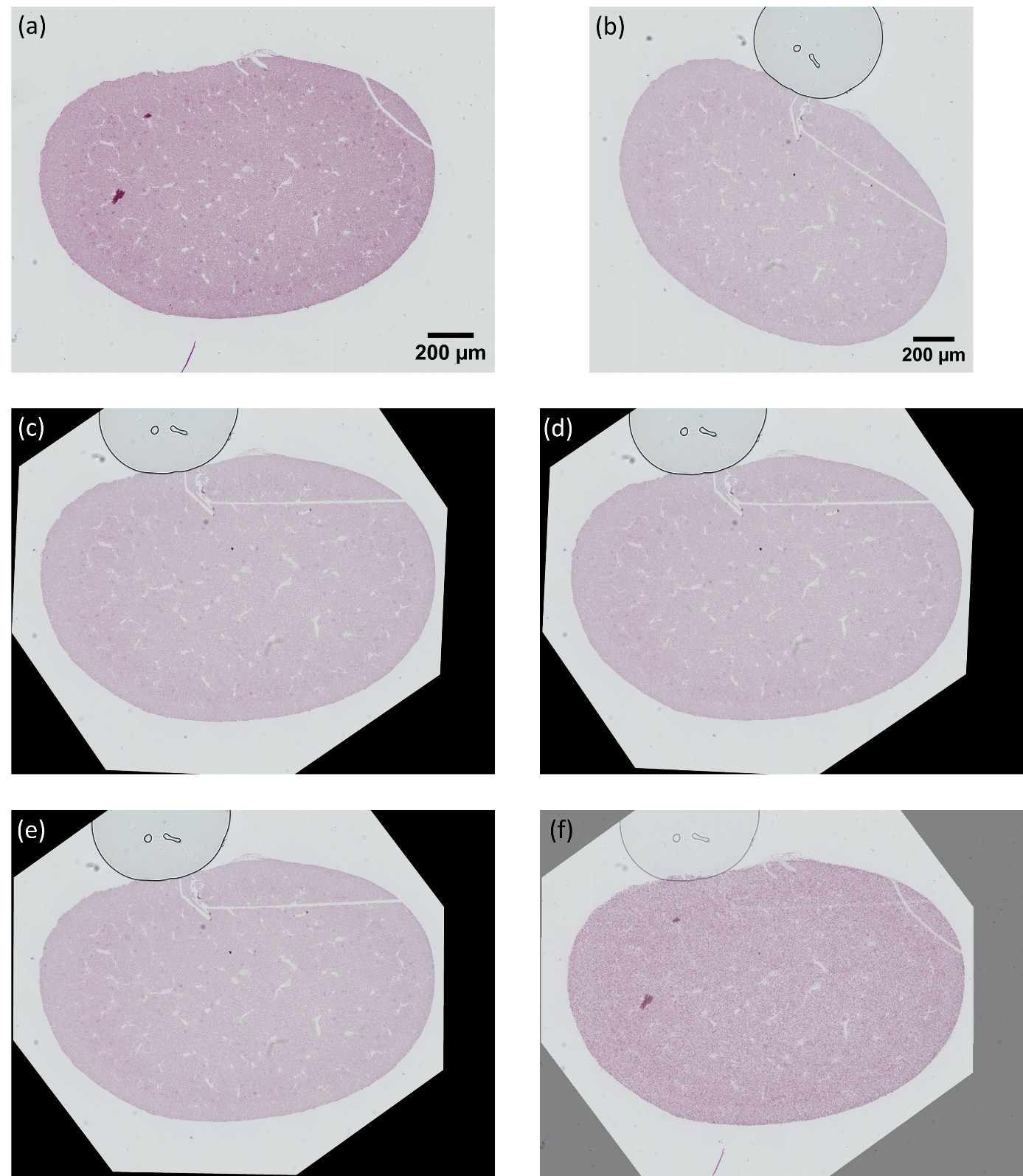


Figure S5. Intensity-based image registration results for the Mammary-gland_1 sample from the ANHIR dataset: (a) Fixed image used as the reference for registration, (b) Moving image to be aligned with the fixed image, (c) Result of the Euler method, (d) Similarity method, (e) Affine method, and (f) Overlay of the Affine method result on the fixed image, demonstrating alignment accuracy.


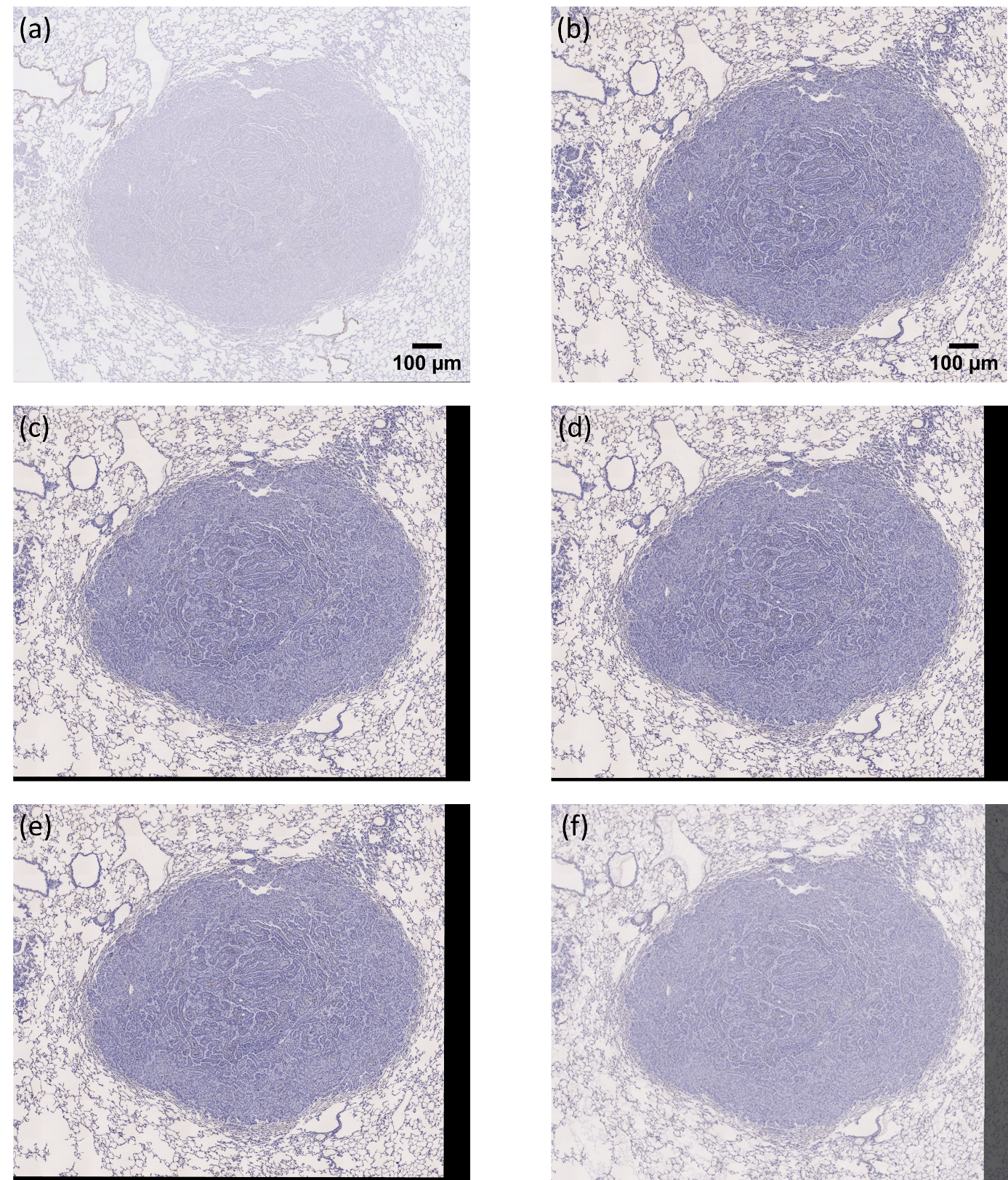


Figure S6. Intensity-based image registration results for the Mammary-gland_1 sample from the ANHIR dataset: (a) Fixed image used as the reference for registration, (b) Moving image to be aligned with the fixed image, (c) Result of the Euler method, (d) Similarity method, (e) Affine method, and (f) Overlay of the Affine method result on the fixed image, demonstrating alignment accuracy.


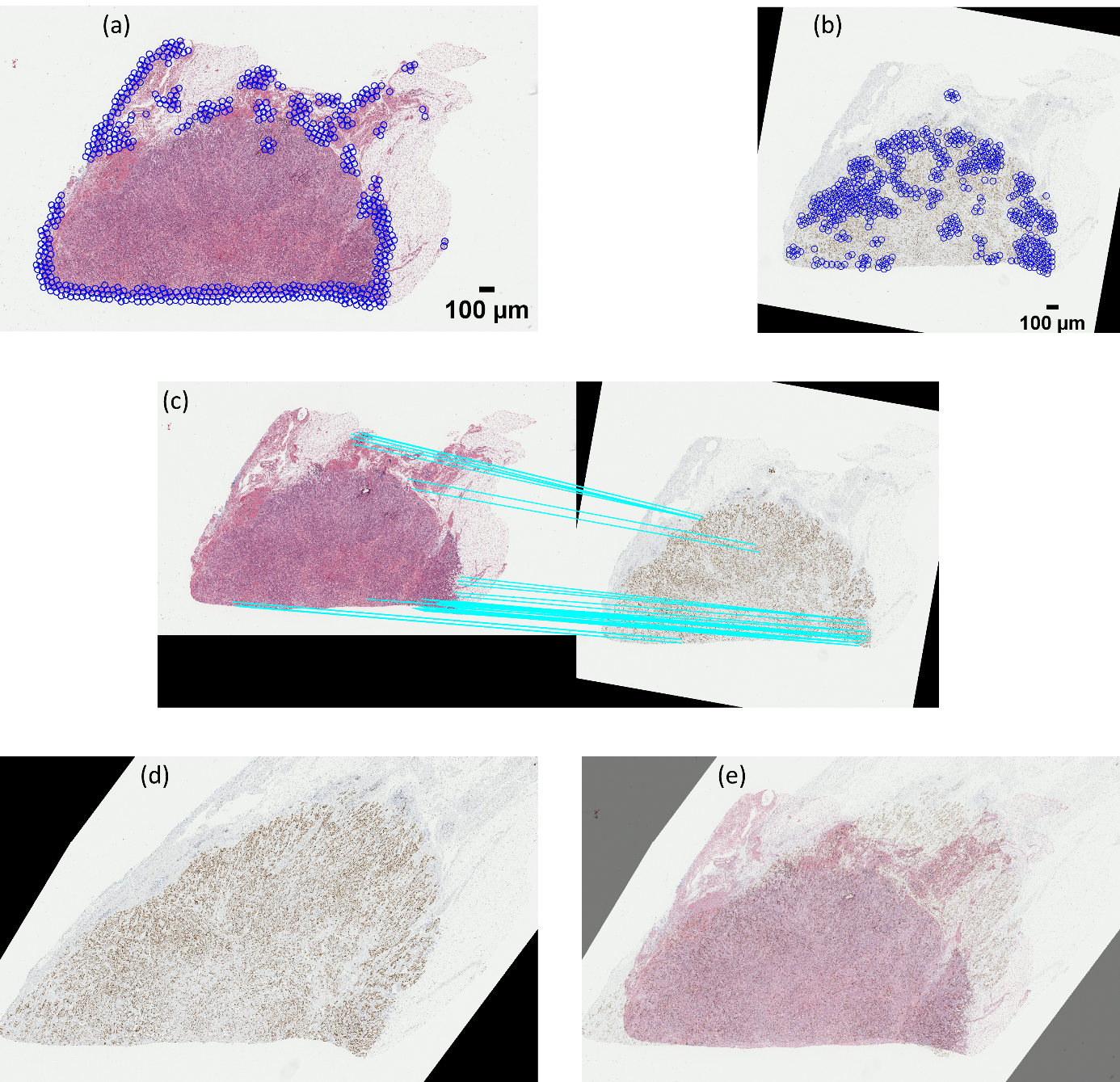
 Figure S7. Results of feature-based image registration using our new approach on the COAD_05 sample. (a) Key points detected in the fixed image, (b) key points detected in the moving image, (c) feature mapping between the two modalities, (d) image registration result using our approach, and (e) overlay of the registered image over the fixed image, demonstrating the accuracy of the alignment.


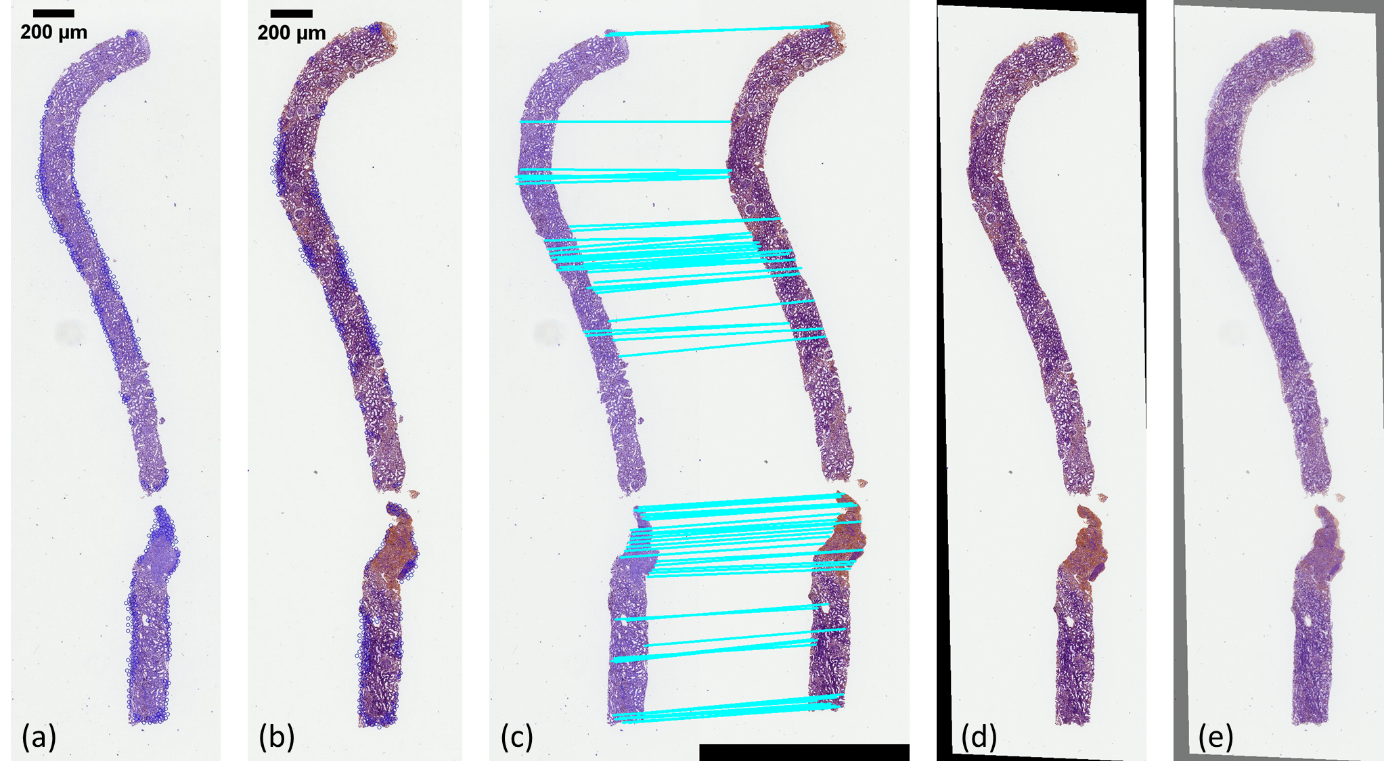
 Figure S8. Results of feature-based image registration using our new approach on the COAD_05 sample. (a) Key points detected in the fixed image, (b) key points detected in the moving image, (c) feature mapping between the two modalities, (d) image registration result using our approach, and (e) overlay of the registered image over the fixed image, demonstrating the accuracy of the alignment.


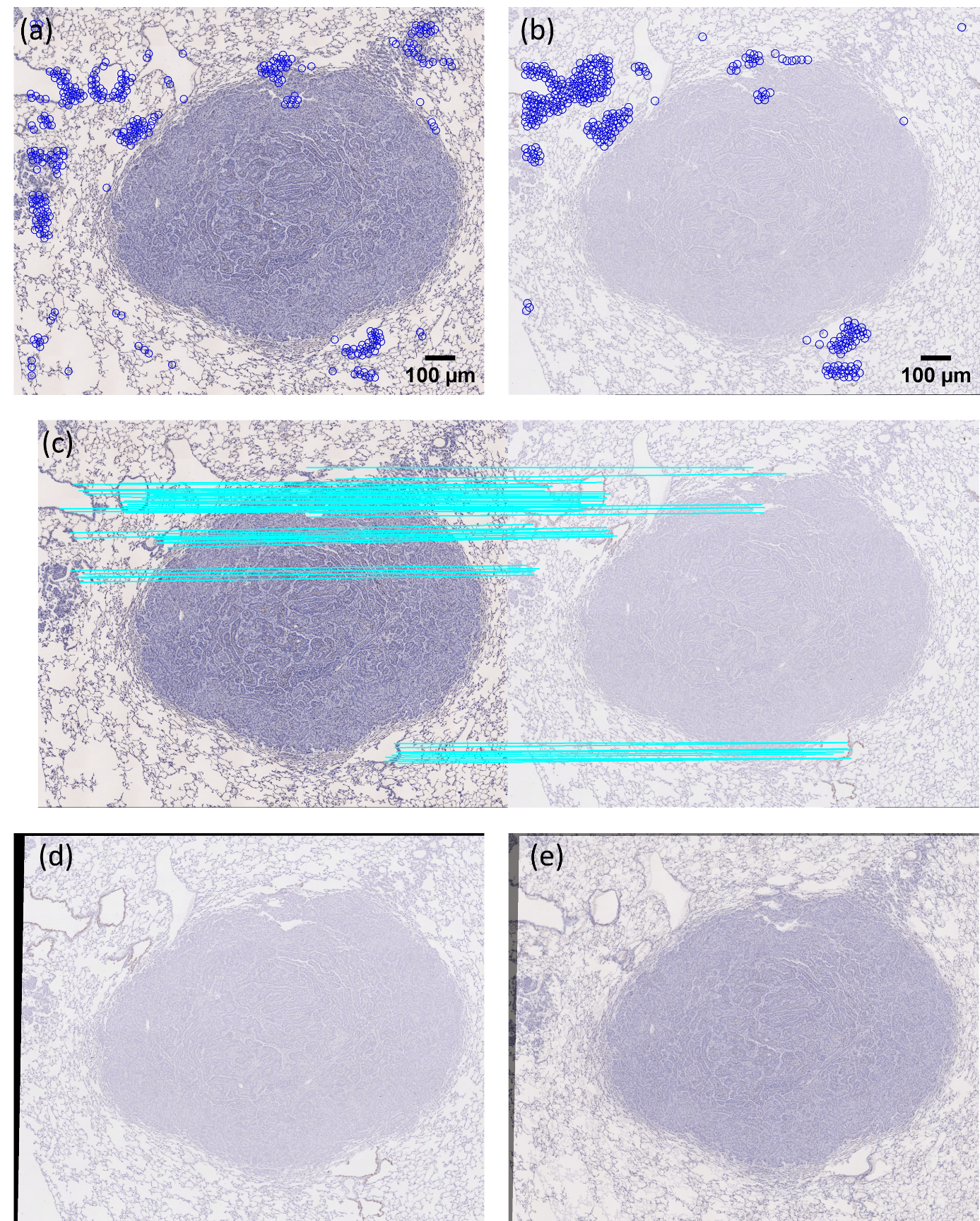
 Figure S9. Results of feature-based image registration using our new approach on the COAD_05 sample. (a) Key points detected in the fixed image, (b) key points detected in the moving image, (c) feature mapping between the two modalities, (d) image registration result using our approach, and (e) overlay of the registered image over the fixed image, demonstrating the accuracy of the alignment.


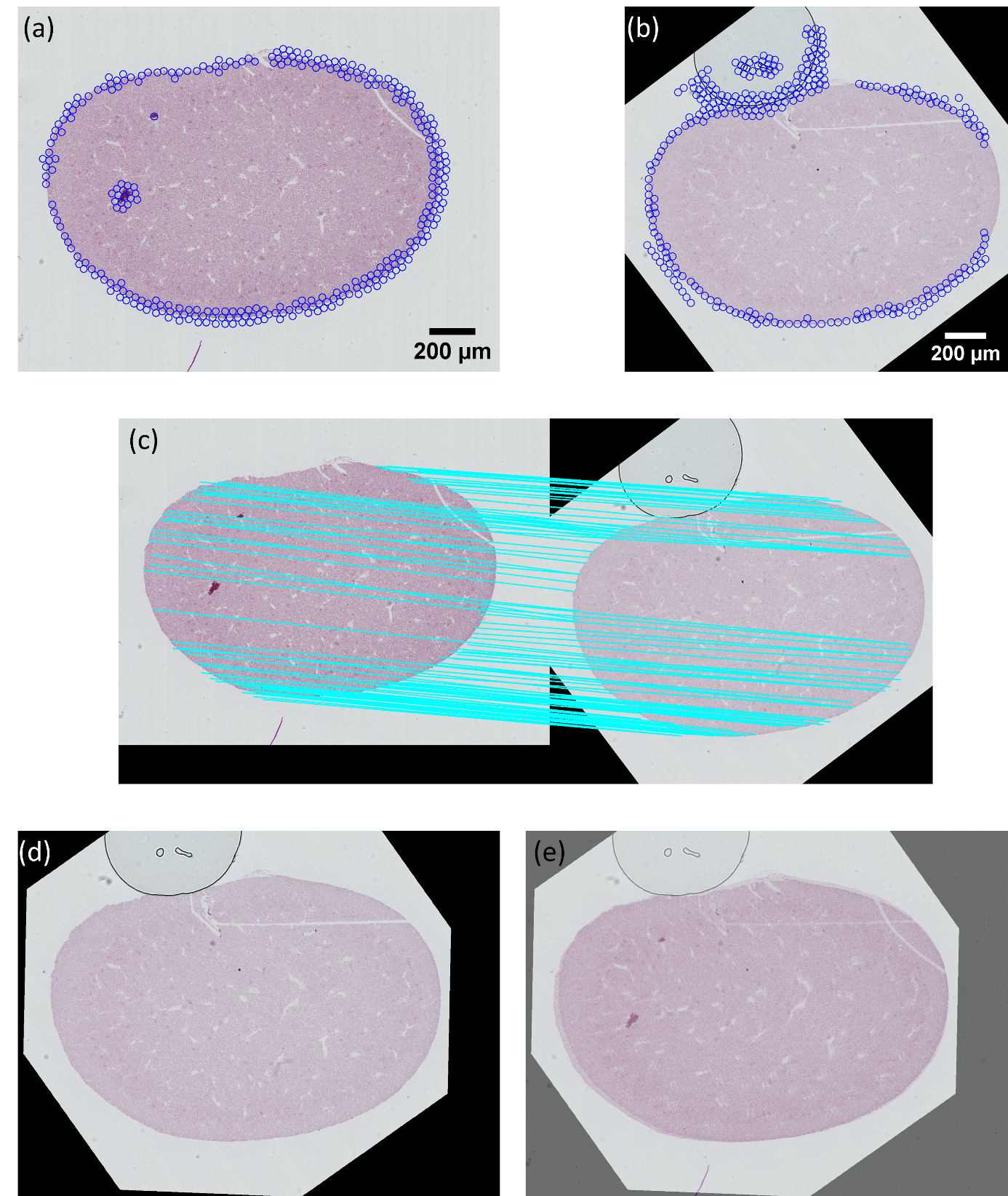
 Figure S10. Results of feature-based image registration using our new approach on the COAD_05 sample. (a) Key points detected in the fixed image, (b) key points detected in the moving image, (c) feature mapping between the two modalities, (d) image registration result using our approach, and (e) overlay of the registered image over the fixed image, demonstrating the accuracy of the alignment.

**References**

1. Klein, S., et al., *Elastix: a toolbox for intensity-based medical image registration.* IEEE transactions on medical imaging, 2009. **29**(1): p. 196-205.
